# Maintenance of Stem Cell Niche Integrity by a Novel Activator of Integrin Signaling

**DOI:** 10.1371/journal.pgen.1006043

**Published:** 2016-05-18

**Authors:** Joo Yeun Lee, Jessica Y. Chen, Jillian L. Shaw, Karen T. Chang

**Affiliations:** 1 Zilkha Neurogenetic Institute, Keck School of Medicine, University of Southern California, Los Angeles, California, United States of America; 2 Neuroscience Graduate Program, University of Southern California, Los Angeles, California, United States of America; 3 Department of Cell and Neurobiology, Keck School of Medicine, University of Southern California, Los Angeles, California, United States of America; University of Northern Colorado, UNITED STATES

## Abstract

Stem cells depend critically on the surrounding microenvironment, or niche, for their maintenance and self-renewal. While much is known about how the niche regulates stem cell self-renewal and differentiation, mechanisms for how the niche is maintained over time are not well understood. At the apical tip of the *Drosophila* testes, germline stem cells (GSCs) and somatic stem cells share a common niche formed by hub cells. Here we demonstrate that a novel protein named Shriveled (Shv) is necessary for the maintenance of hub/niche integrity. Depletion of Shv protein results in age-dependent deterioration of the hub structure and loss of GSCs, whereas upregulation of Shv preserves the niche during aging. We find Shv is a secreted protein that modulates DE-cadherin levels through extracellular activation of integrin signaling. Our work identifies Shv as a novel activator of integrin signaling and suggests a new integration model in which crosstalk between integrin and DE-cadherin in niche cells promote their own preservation by maintaining the niche architecture.

## Author Summary

Stem cells are vital for development and for regeneration and repair of tissues in an organism. The ability of adult stem cells to maintain their “stemness” depends critically on the localized microenvironment, or niche. While much is known about how the niche regulates stem cell self-renewal and differentiation, mechanisms for how the niche is maintained during aging are not well understood. Using Drosophila testis as a model system, here we demonstrate that a protein we named Shriveled is a secreted protein that activates integrin signaling to preserve niche architecture. We also show that Shriveled-dependent activation of integrin maintains normal E-cadherin levels in the niche cells, providing a mechanism for niche maintenance. Interestingly, upregulation of Shriveled retards the loss of niche and stem cells seen during normal aging. Together, our work identifies Shriveled as a novel molecule required for preservation of the niche structure in the *Drosophila* testis.

## Introduction

Adult stem cells have the unique capacity to undergo self-renewal for extended periods of time and to generate differentiating daughter cells with the potential for tissue repair and regeneration. Such features of adult stem cells depend critically on the microenvironment, or niche [1]. The stem cell niche—comprised of various molecular factors such as extracellular matrix (ECM), secreted proteins, adhesion molecules and support cells—provides the key molecular cues necessary for stem cell maintenance and tissue homeostasis during development, aging and changes in environment [1–5]. Despite a wealth of knowledge on how the niche-stem cell interactions control their self-renewal and differentiation, mechanisms for how the niche is maintained over time are not well understood.

The *Drosophila* germline stem cell system is an excellent model system for investigating the biology of stem cells *in vivo* in the context of their niche, mainly due to its simple anatomy and easily identifiable stem cell populations [6–10]. In *Drosophila* testes, germline stem cells (GSCs) and cyst stem cells (CySCs) share a common niche formed by hub cells. Each hub contains roughly 10 somatic hub cells located at the apical tip of the testes and is surrounded by ~ 6–10 GSCs [11–13]. Each GSC is also enveloped by two CySCs that are also in direct contact with the apical hub. Dynamic signaling between hub cells, CySCs, and GSCs facilitate the self-renewal, differentiation, and survival of the GSCs [6,7,14]. It was shown that hub cells secrete molecules such as Unpaired and Bone morphogenic protein ligands to neighboring stem cells to govern stem cell self-renewal and maintenance [13,15–23].

The molecular cues that regulate stem cell self-renewal and differentiation are short-range signals acting on adjacent somatic and stem cells; therefore, adhesive forces are necessary to anchor hub cells to the appropriate location in the testis, and stem cells to the hub [5,24,25]. Two types of cell adhesion molecules have been shown to serve such functions in the *Drosophila* germline: integrins and cadherins [19,26–33]. Integrins are heterodimeric transmembrane receptors that can signal bi-directionally across the plasma membrane to mediate cell-ECM adhesion [34,35]; cadherins mediate cell-cell adhesions via homophilic interactions of the extracellular domains [36]. In the *Drosophila* male germline system, integrins are crucial for anchoring the somatic hub cells to the basal lamina at the tip of the testis [26], whereas DE-cadherin is required for attaching the GSCs and CySCs to the hub [19,28,29,37]. Altered integrin signaling affects niche positioning and leads to loss of both hub and stem cell populations in the adult testes [26,27,38], thus underscoring the importance of hub cell anchoring in the maintenance of its neighboring stem cells. DE-cadherin and integrin also sustain the “competitiveness” of GSCs and CySCs [13,30,39,40], respectively, although the role of integrin in niche competition is less clear. In the fly testes, expression of a dominant negative construct of DE-cadherin caused GSC loss only if it was expressed in a subset of GSCs, but not if in all GSCs, demonstrating DE-cadherin influences competition between GSCs [39]. It has also been shown that CySCs with elevated levels of βPS integrin at the hub-CySC interface caused by loss of Socs36E out-compete the GSCs and displace them away from the niche [13]. However, more recent data suggest that loss of Soc36E does not elevate integrin levels but activates MAPK to increase competitiveness of CySCs, and that clonal overexpression of integrin in CySCs does not cause niche competition [41,42]. Despite current controversial results on the role of integrin in niche competition, previous work on mechanisms maintaining GSC and stem cell niche have suggested that optimum integrin and DE-cadherin signaling are crucial for a healthy stem cell system.

Here we identify a novel activator of integrin signaling named Shriveled (Shv) in the maintenance of stem cell niche integrity in the *Drosophila* testes. We report that Shv is secreted extracellularly by somatic cells and GSCs to activate βPS integrin *in vivo* to ensure anchoring of the hub cells and maintenance of niche architecture. Importantly, our results indicate Shv modulates DE-cadherin levels through an integrin-dependent pathway, thus uncovering a new integration mechanism in which crosstalk between integrin and DE-cadherin in hub cells serves to maintain niche architecture and GSC numbers. Furthermore, our findings that upregulation of Shv preserves the stem cell niche in aging *Drosophila* males further implies that enhancing Shv function may be a valuable strategy to strengthen adhesion within the niche in order to delay the effects of aging on tissue homeostasis.

## Results

### Deterioration of hub architecture in shriveled mutants

**Fig 1:**
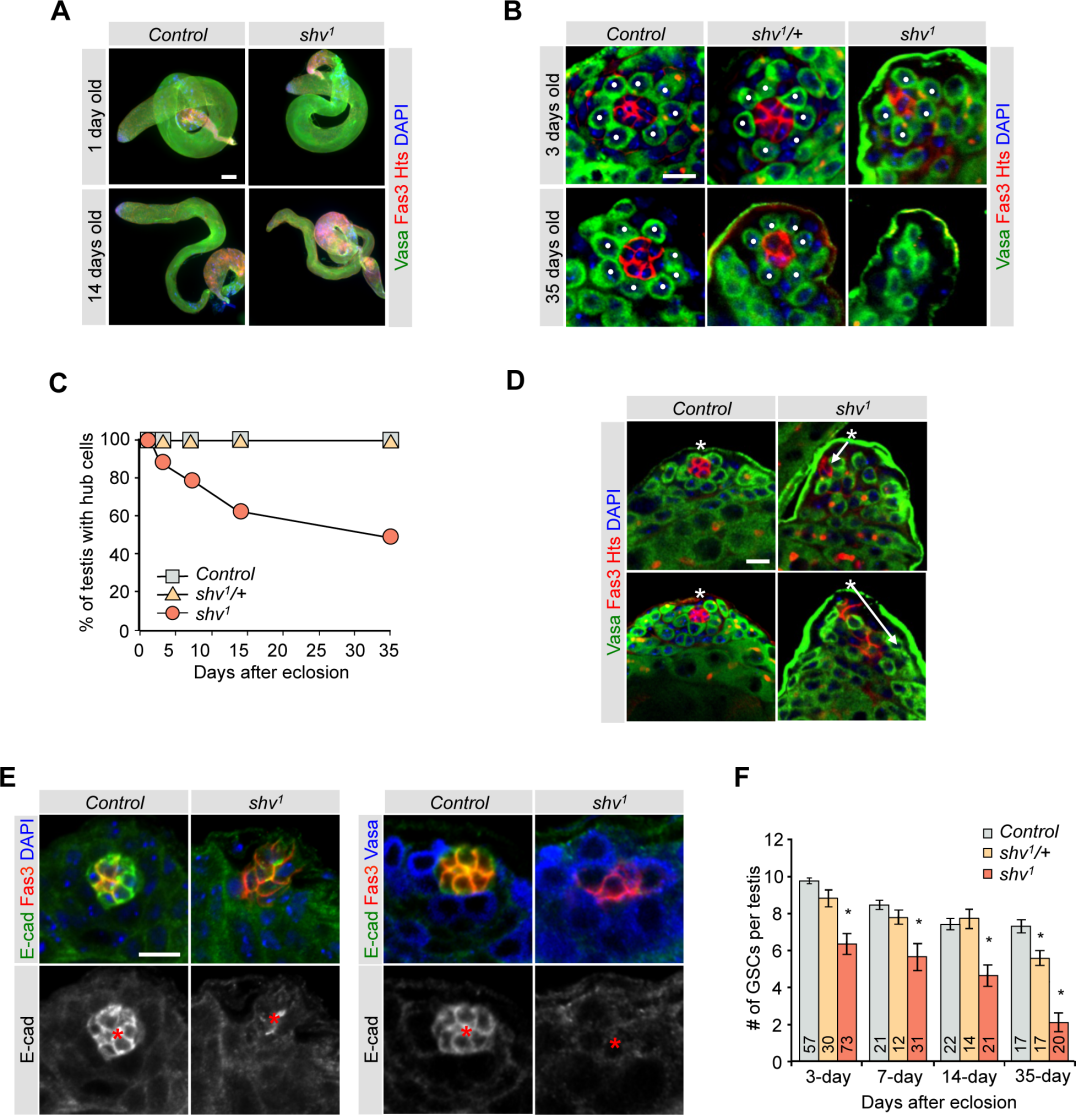
Shv is required for the maintenance of hub and GSCs during aging. (A) Representative images of testes dissected from control and *shv*^*1*^ flies at the indicated age. Scale bar = 100 μm. (B) Magnified images of 3 and 35 days old testes from control, *shv*^*1*^/+ and *shv*^*1*^ flies immunostained with antibodies as indicated. Dots highlight GSCs. (C) Quantification of percentage of testes with hub cells. Sample numbers are indicated in Table 1. (D) Representative confocal images (from a single Z-axis plane) demonstrating hub architecture of young control and *shv*^*1*^ testes. Asterisks show the apical tip of the testis and arrows highlight hub mislocalization. (E) Representative images of the testis tip stained with the indicated antibodies in control and *shv*^*1*^. * highlights the hub. For (B), (D), and (E) scale bar = 10 μm. (*F*) Quantification of average number of GSCs per testis during aging. Sample numbers are indicated in the bar graph. Age of flies examined is 3 days after eclosion unless noted otherwise. For multiple samples, One-way ANOVA followed by post hoc analysis with Bonferroni’s multiple-comparison test was used to determine statistical significance. * p < 0.05 compared to control. All values represent mean ± SEM.

We isolated a *Drosophila* mutant that displayed male sterility and age-dependent decrease in testes size (Fig 1A). Because of the shrinking testes phenotype, we named the mutant *shriveled* (*shv*) and the allele *shv*^*1*^. To understand the cause of the reduced testes size, we stained them with a hub marker Fasciclin III (Fas III) and germ cell marker Vasa. *shv*^*1*^ mutants showed a striking age-dependent loss of the hub structure (Fig 1B and 1C). By 35 days of age, about 50% of the *shv*^*1*^ testes showed a complete loss of hub cells while all of the control and *shv*^*1*^/+ testes still contained a hub. We also counted the average number of hub cells during aging (Table 1), and found that *shv*^*1*^ mutant showed a slight decrease in the number of hub cells at day 3 (control: 8.9 ± 0.18 vs. *shv*^*1*^: 6.4 ± 0.28; p < 0.05). By 35 days of age, the average number of hub cells is 8.18 ± 0.26 for control, but 1.8 ± 0.46 for *shv*^*1*^ (or 4.0 ± 0.4, n = 16 if only counting testes that contained positive hub marker staining). To further understand if *shv*^*1*^ is required for normal hub formation, we counted the number of hub cells in 3^rd^ instar larval testes. *shv*^*1*^ larval testes showed a normal number of hub cell compared to control (S1A Fig), suggesting that Shv is not essential for hub formation, but rather required for hub maintenance. Closer examination of the hub architecture of young flies revealed that even though *shv*^*1*^ mutants contained a Fas III-positive hub, 58.3% of hub cells appeared to be “pulled”, spread throughout different image planes of the testes, and frequently did not localize at the apical tip (Figs 1D, S1B and S1C, S1 and S2 Videos). The age-dependent loss of hub cells prompted us to examine the expression level of DE-cadherin, a cell adhesion molecule highly expressed in the hub and previously shown to decline during aging [12,43]. *shv*^*1*^ testes displayed a decrease in DE-cadherin levels in both the hub and hub/GSC border. Both DE-cadherin and DN-cadherin have been shown to be important for niche-GSC interaction [44], we therefore also examined the levels of DN-cadherin in *shv*^*1*^. We found that *shv*^*1*^ testes also showed a decrease in the level of DN-cadherin in the hub cells (S1D Fig). To further confirm that the decrease in cadherins is not due to a loss of hub cell integrity, we examined the level of another hub selective marker, cactus [20]. Similar to Fas III staining, cactus staining remained intact in *shv*^*1*^, suggesting that decrease in DE-cadherin and DN-cadherin levels is not simply due to a loss of hub cell integrity (Fig 1E and S1E Fig).

**Table 1:** Hub cell quantification. Table showing quantification of hub cell numbers and number of testes quantified. Each column represents designated days after eclosion. Sample numbers are in parentheses.

Genotype	3 DAE	7 DAE	14 DAE	35 DAE	Total (n)
*Control*	8.9 ± 0.18 (106)	8.0 ± 0.18 (20)	8.1 ± 0.29 (21)	8.2 ± 0.26 (17)	164
*shv*^*1*^*/+*	8.8 ± 0.25 (33)	9.3 ± 0.45 (12)	8.9 ± 0.61 (14)	8.2 ± 0.25 (17)	76
*shv*^*1*^	6.4 ± 0.28 * (134)	5.8 ± 0.63 * (31)	5.1 ± 0.70 * (22)	1.8 ± 0.47 * (30)	217
*upd-GAL4;shv*^*1*^*;UAS-shv*	2.9 ± 1.15 * (11)	2.9 ± 1.17 * (15)	2.4 ± 1.56 * (8)	0.0 ± 0.00 * (10)	44
*upd-GAL4/UAS-shv*	8.1 ± 0.36 (15)	7.8 ± 0.22 (9)	7.1 ± 0.30 (8)	7.0 ± 0.44 (7)	39
*shv*^*1*^*;UAS-shv/C833-GAL4*	9.8 ± 0.44 (26)	8.4 ± 0.57 (20)	7.9 ± 0.51 (20)	6.0 ± 0.98 (23)	89
*UAS-shv/C833-GAL4*	9.3 ± 0.24 (23)	8.4 ± 0.40 (12)	7.5 ± 0.29 (20)	7.3 ± 0.27 (20)	75
*shv*^*1*^*;UAS-shv/nanos-GAL4*	7.4 ± 0.37 * (33)	6.4 ± 0.70 * (14)	6.2 ± 0.82 * (10)	3.5 ± 0.71 * (20)	77
*UAS-shv/nanos-GAL4*	9.8 ± 0.24 * (19)	8.4 ± 0.26 (12)	8.4 ± 0.27 (10)	7.8 ± 0.33 (10)	51

* p < 0.05 compared to control in the same age group. For multiple samples, One-way ANOVA followed by post hoc analysis with Bonferroni’s multiple-comparison test was used to determine statistical significance.

Studies have shown the CySCs, together with hub cells, form part of the niche for GSCs. We therefore determined if *shv*^1^ mutants also have altered CySCs by counting the number of Zfh1-positive, Eya-negative cells [45]. S1F Fig shows that there is also a slight but significant decrease in the number of CySCs. Accordingly, s*hv*^*1*^ testes have reduced number of GSCs (Fig 1F). Together, our results demonstrate that Shv is essential for the maintenance of niche integrity and GSCs.

### Shriveled encodes a conserved protein

**Fig 2:**
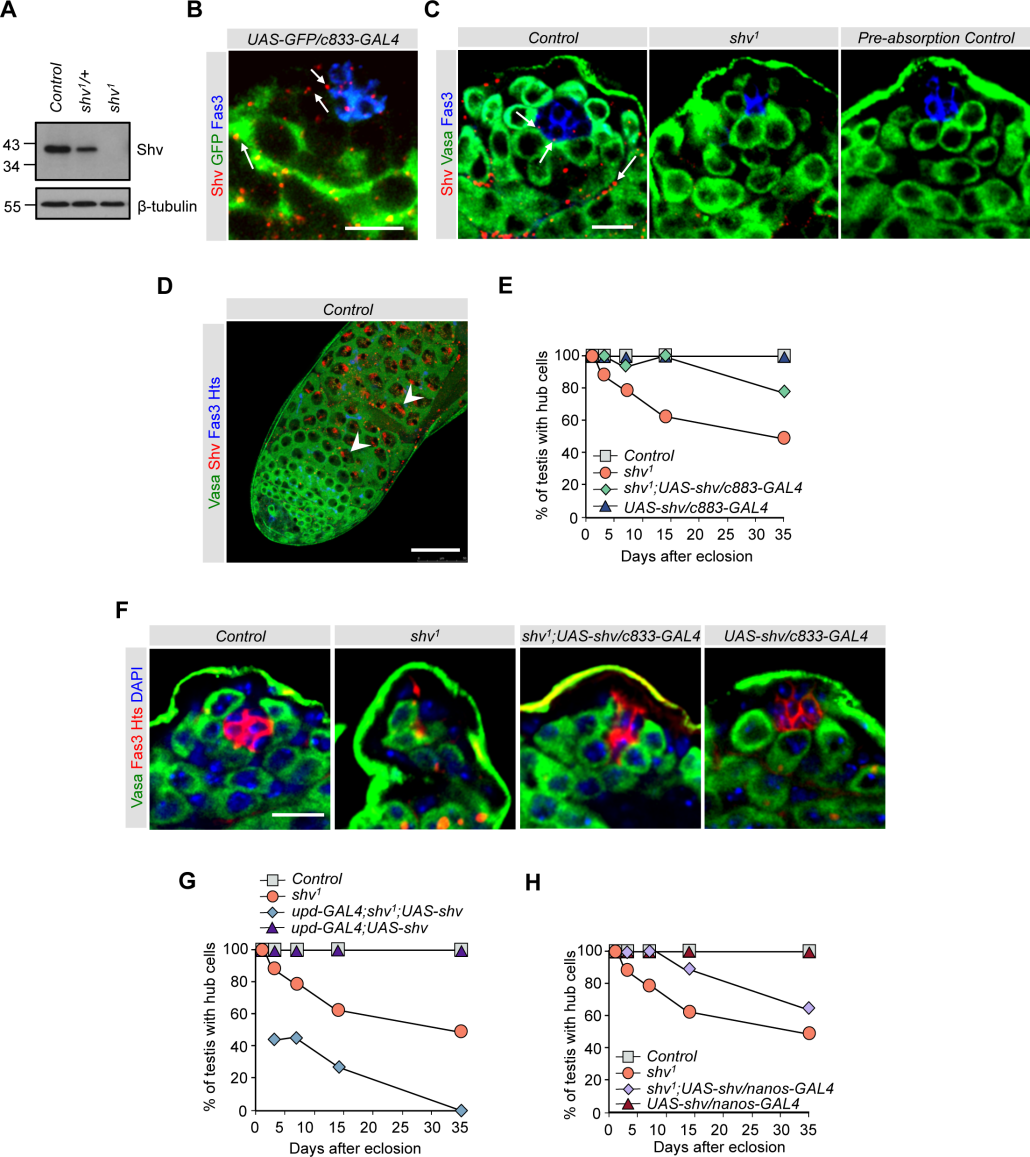
Shv is present and required in multiple cell types. (A) Representative western blot showing Shv levels for the indicated genotypes. β-tubulin is used as a loading control. (B) Testes immunostained with the indicated antibodies. Arrows point to Shv staining often seen at the hub/GSC, GSC/CySC, and germ/cyst interface. (C) *shv*^*1*^ mutant displays a significant decrease in Shv level. Antibody pre-absorbed with Shv peptide confirms Shv antibody specificity. Arrows point to Shv present at hub/GSC interface. (D) Control testes taken at lower magnification shows abundant Shv protein in the nucleus of spermatocytes (arrowhead). Scale bar = 50 μm. For (B), (C), and (D), Images of testes are from 3 day old males. (E), (G), and (H) Quantification of percentage of testes with hub cells for the indicated genotypes. (F) Images of testes from 14 day old flies showing rescue of the hub phenotype in flies overexpressing *shv* using *C833-GAL4* driver in *shv*^*1*^ mutant background. In (B), (C), and (F), scale bar = 10 μm and sample number per age group is listed in Table 1.

Molecular cloning identified that *shv*^*1*^ contains P-element insertion within an uncharacterized fly gene, CG4164, which shares 63% identity in amino acid sequence with human DNAJB11 (S2A Fig). Human DNAJB11, also known as ERDJ3, is a unique chaperone protein that contains a signal peptide sequence at the N-terminus, a putative nuclear localization signal, two DnaJ domains and a RGD motif found in some integrin binding proteins [46–48]. It has been shown to act as a co-chaperone in assisting proper protein folding in the ER and in increasing the activity and affinity of Hsp70 BiP for substrates [47,49,50]. It also acts as a secreted protein that modulates integrin affinity [51] and in modulating unfolded protein response [52]. However, roles of DNAJB11 in stem cell maintenance and spermatogenesis have not been established. Quantitative RT-PCR revealed that the *shv*^*1*^ mutant is nearly a null allele with *shv* transcript level of only about 0.025 ± 0.002 fold of the control, and an undetectable level of Shv protein on Western blot using an antibody specific for Shv (Figs 2A, S2B and S2C). Quantitative RT-PCR also confirmed that *shv*^*1*^ does not affect the transcription level of crq, an adjacent gene oriented in the opposite direction (S2A and S2B Fig). To further confirm that mutation in Shv is responsible for the observed phenotypes, we isolated a precise excision allele, *shv*^*PJ*^, which reverted the *shv* transcript level back to wildtype, as well as the positioning and structure of the hub, number of GSCs and DE-cadherin level to normal (S3 Fig). In addition, we obtained other alleles of Shv with transposon insertion within CG4164, *shv*^*9803*^ and *shv*^*c00496*^ (S2A Fig). Note that we did not characterize *shv*^*9803*^ homozygous mutant because it is a strong semi-lethal allele. Nevertheless, similar to *shv*^*1*^, *shv*^*9803*^*/shv*^*1*^, *shv*^*c00496*^, *and shv*^*c00496*^*/shv*^*1*^ also showed abnormal hub architecture, DE-cadherin reduction and loss of GSCs (S3 Fig), but did not show a complete loss of hub with aging. Quantitative RT-PCR revealed that the difference is likely caused by *shv*^*c00496*^ and *shv*^*9803*^ being less severe alleles of *shv*^*1*^ (S3A Fig). Together, these data suggest that reduction in Shv level is responsible for the hub phenotypes and reduction in GSC number.

To elucidate how Shv maintains the stem cell niche, we examined its distribution in the fly testes. Fluorescent i*n situ* hybridization revealed that *shv* RNA is ubiquitously expressed in different cell types: low levels in hub cells, CySCs and GSCs; high levels in spermatocytes and cyst cells; below the level of detection in *shv*^*1*^ mutant (S4A Fig). Immunostaining with the Shv antibody further showed low levels of Shv protein in hub cells and CySCs at the apical tip of the wildtype testes, but barely detectable in *shv*^*1*^ mutants or not present if the antibody had been pre-absorbed with the Shv immunizing peptide (Figs 2B, 2C and S2C). These results demonstrate the specificity of the Shv antibody and further confirm that *shv*^*1*^ is close to a null allele. Note that because Shv staining often appeared punctate at the apical tip, we performed additional colocalization studies using specific organelle markers. S4B and S4C Fig show that Shv displayed partial colocalization with the ER, but not with spectrosome, peroxisome, lysosome, mitochondria, or golgi markers in cyst cells. Instead, Shv is frequently seen at the GSC/hub interface and cyst/germ cell border (Fig 2B and 2C; arrows), thus making it difficult to specifically locate in a specific cell type. These staining patterns are characteristics of a secreted protein, and the presence of a signal peptide sequence at the N-terminus of Shv further suggests that Shv may be a secreted protein. We also noted that despite *shv* RNA being present in the GSCs, Shv protein is usually below the level of detection in GSCs. In addition, consistent with the presence of a predicted nuclear localization signal in the Shv protein (S2A Fig), Shv was detected in the nucleus of primary spermatocytes, but not in *shv*^*1*^ mutant (Fig 2D and S4D Fig). The presence of Shv in different cell types suggests that Shv protein may play multiple roles in spermatogenesis.

To identify if Shv is required in specific cell types to maintain hub integrity and preserve GSC number, we knocked down Shv in selective cells using different driver and UAS-RNAi lines, but were not able to detect any phenotype likely because we were not able to sufficiently reduce Shv level. We therefore restored Shv protein in *shv*^*1*^ testes using the UAS/GAL4 system and independent driver lines specific for expression in hub cells (*upd-GAL4*) [44], germ cells (*nanos-GAL4*) [53], and the hub + cyst cells (*c833-GAL4*) [54,55]. Expression of Shv using *c833-GAL4* rescued both hub integrity and GSC number (Figs 2E, 2F and S5A). Note that all early somatic drivers we tested show low expression in the hub, including the commonly used *c587-GAL4*, *ptc-GAL4*, *Tj-GAL4*, and *esg-GAL4* [56–58]. To confirm that this rescue is not driver specific, we also performed the rescue experiment with *C587-GAL4* driver. Similarly, expressing Shv in *shv*^*1*^ mutant using *C587-GAL4* restored the number of hub cells (S5B Fig) and hub mislocalization phenotype (0% hub mislocalization for *c587-GAL4; shv*^*1*^*; UAS-Shv*). Expression of full-length Shv in hub cells of *shv*^*1*^ mutants not only failed to rescue the age-dependent loss of hub, but also exacerbated the phenotype (Fig 2G and Table 1). It is possible that abnormally high level of Shv locally during development may interfere with hub formation, since the rescue experiment using *upd-GAL4* driver had a dominant effect over *shv*^*1*^ mutants. Notably, even though Shv is below the level of detection in GSCs by immunostaining, expression of full-length Shv in *shv*^*1*^ germ cells using *nanos-GAL4* also partially preserved the hub and protected against decreases in GSC number (Fig 2H, Table 1, and S5C Fig). Together, our results indicate that expression of Shv in either somatic cells or GSCs could maintain hub integrity and GSC health via both cell-autonomous and non-cell-autonomous mechanisms.

### Shriveled interacts with integrin to control hub anchoring

**Fig 3:**
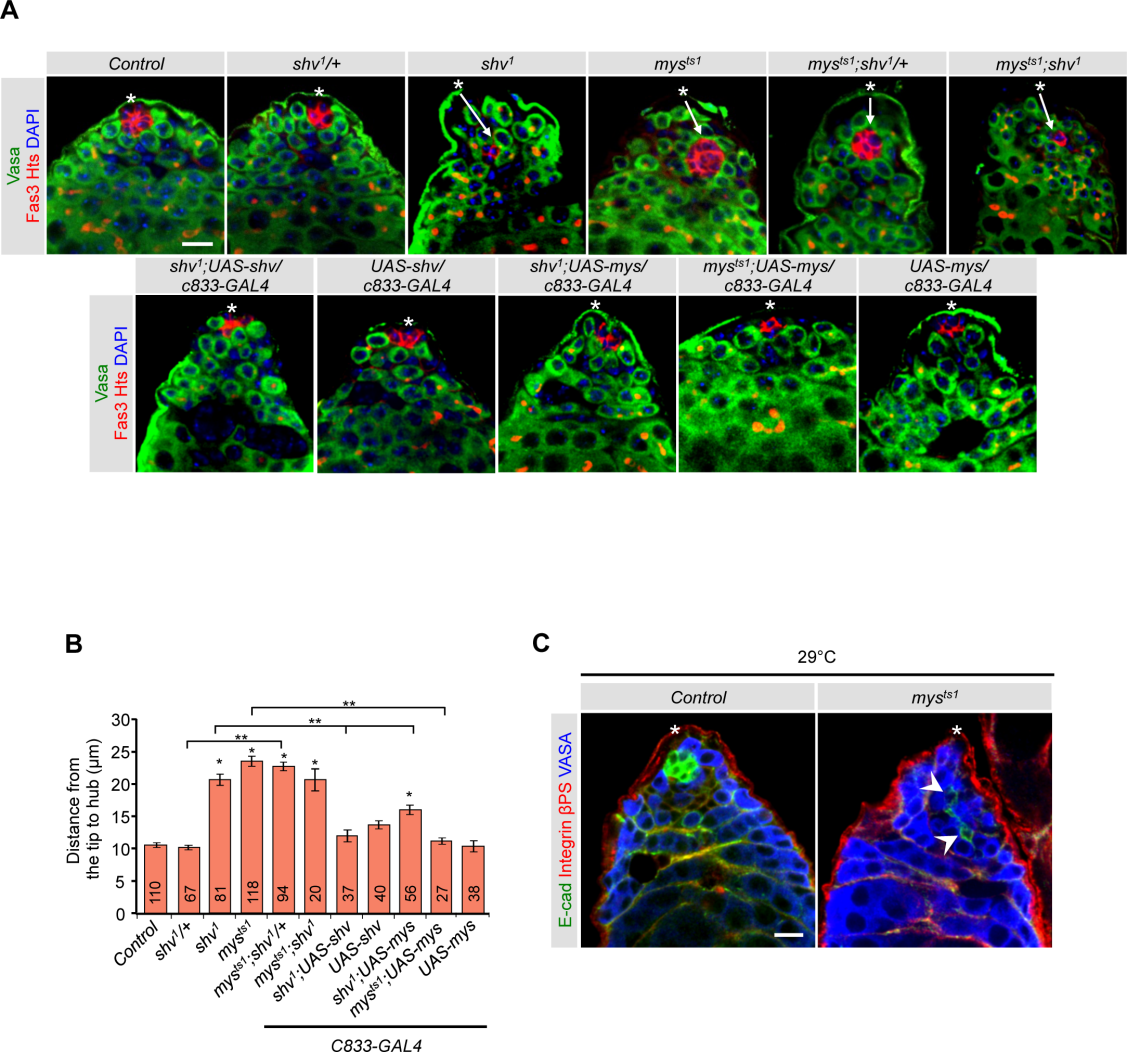
Shv genetically interacts with βPS integrin signaling. (A) Representative images of testes dissected from 3 day old flies reared at 25°C. Asterisks show the apical tip of the testis and arrows highlight distally located hub. (B) Quantification of hub position relative to the apical tip of the testis. * p < 0.05 compare to control and ** p < 0.05 compared to the indicated genotypes. All values represent mean ± SEM and sample numbers are indicated in the bar graph. (C) Testes of *mys*^*ts1*^ raised at 29°C show similar hub phenotype as seen in *shv*^*1*^. Arrowheads highlight the “pulling” of hub cells and asterisks indicate the apical tip. Note that DE-cadherin staining is also reduced in *mys*^*ts1*^ raised at 29°C. Age of flies examined is 3 days after eclosion unless noted otherwise. For multiple samples, One-way ANOVA followed by post hoc analysis with Bonferroni’s multiple-comparison test was used to determine statistical significance. Scale bar in (A) and (C) = 10 μm.

Integrin is an essential cell adhesion molecule found in somatic cells of *Drosophila* testes. Loss of integrin signaling causes the hub to drift away from the apical tip, and in severe cases, leads to loss of hub cells as seen in *talin-RNAi* lines [26,27,38]. The hub mislocalization and loss of hub phenotypes seen in *shv*^*1*^ mutants are similar to those described for βPS integrin mutants; we therefore tested the possibility that Shv interacts with integrin signaling pathway. We first assayed for genetic interaction between Shv and βPS integrin, *myospheroid* (*mys*) in *Drosophila* [59], by measuring the hub to testes tip distance. Since null alleles of *mys* are embryonic lethal due to muscle detachment [60], we used a viable *mys* hypomorphic allele (*mys*^*ts1*^) that had been shown to decrease levels of βPS integrin when raised at 25°C [61]. *mys*^*ts1*^ testes indeed showed hub mislocalization that was rescued by *mys* overexpression in somatic cells. Similarly, hub in *shv*^*1*^ was mislocalized but was rescued by Shv upregulation (Fig 3A and 3B), indicating Shv is responsible for the phenotype. Double mutants of *mys*^*ts1*^*; shv*^*1*^ showed the same hub mislocalization phenotype as either mutant alone, suggesting that Shv and Mys act in the same pathway to ensure normal anchoring of the hub. Upregulation of Mys in *shv*^*1*^ mutant background partially restored the hub mislocalization phenotype (Fig 3A and 3B), confirming genetic interaction between the two proteins. However, the weak rescue further raises the possibility that Shv is required for effective integrin activation.

Curiously, even though *mys*^*ts1*^ exhibited hub mislocalization, the overall hub structure was not altered. This suggests that either there is still sufficient level of functional integrin receptors, or that Shv acts through integrin-independent pathways to influence hub architecture. To further reduce integrin, we reared the *mys*^*ts1*^ flies at 29°C, which resulted in semi-lethality. However, in addition to showing hub mislocalization, hub cells from the testes of surviving *myst*^*s1*^ flies often exhibited a similar mis-aggregation phenotype of hub as seen in *shv*^*1*^ mutants (Fig 3C; 75% of testes examined). These results suggest that integrin, in addition to anchoring the hub at the tip, is required for the maintenance of hub architecture.

### Shriveled is a secreted protein that activates integrin signaling *in vitro*

**Fig 4:**
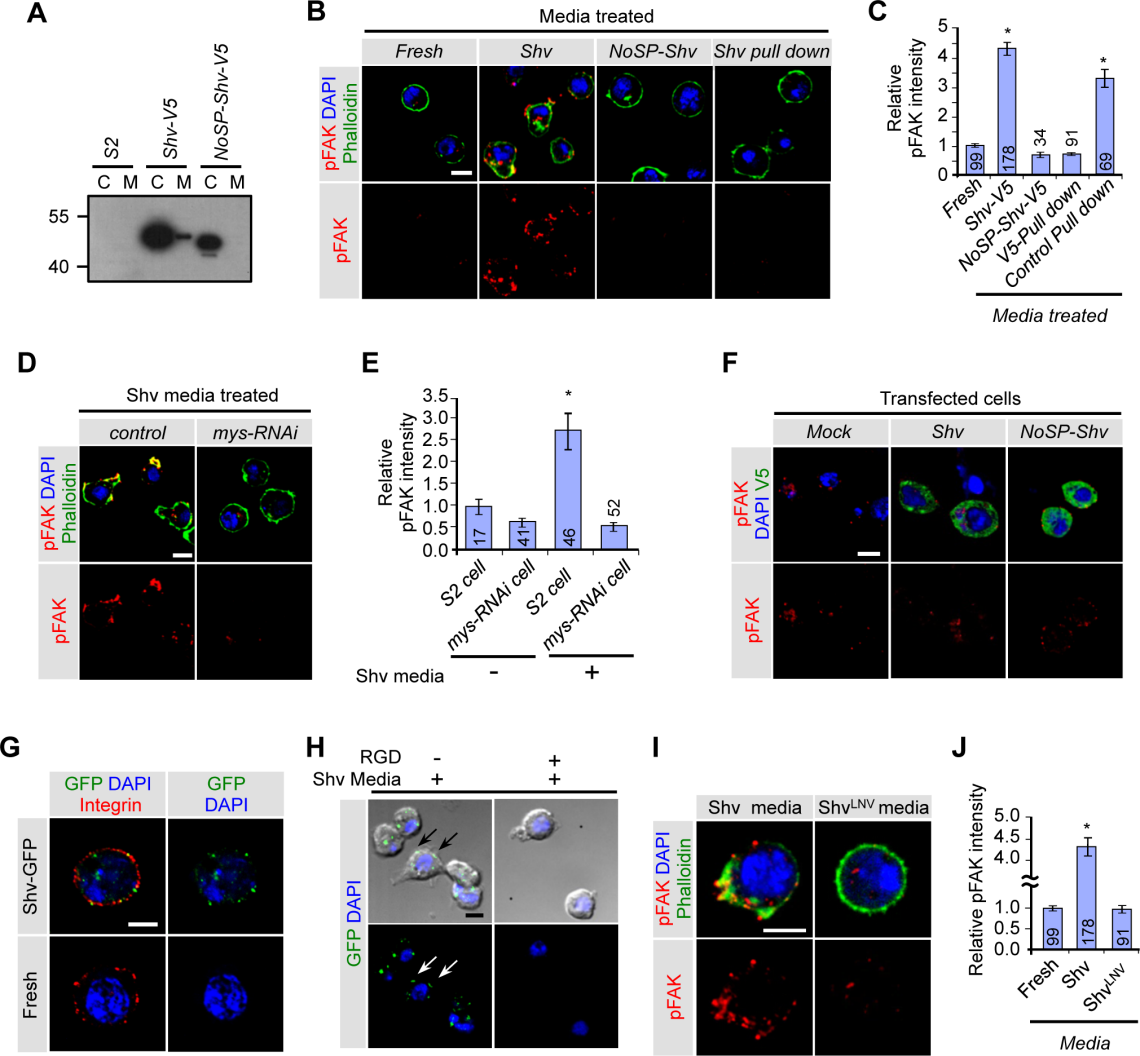
Shv is a secreted protein that activates βPS integrin through outside-in signaling. (A) Western blot showing presence of Shv-V5 protein in the media of transfected cells but not if the signal peptide was truncated. C: transfected cell extract; M: media collected from the transfected cells. (B) Untransfected S2 cells after treatment with the indicated media. Phalloidin (green) staining shows that Shv containing media caused spreading or changes in cell shape and increase in pFAK staining intensity. (C) Quantification of pFAK levels after media treatment. * p < 0.05 compared to cells incubated with fresh media. (D) Images of control and *mys-RNAi* transfected cells following treatment with Shv containing media. Shv containing media failed to induce cell spreading and phosphorylation of FAK when integrin is knocked down. (E) Quantification of relative pFAK intensity. * p < 0.05 compared to control. (F) S2 cells transfected with the indicated constructs but incubated with fresh S2 media did not show changes in pFAK staining. (G) Shv-GFP partially colocalizes with βPS integrin on the S2 cell surface. S2 cells were incubated with either Shv-GFP containing media or fresh S2 media only and processed for staining without permeabilization. (H) Images of S2 cells pre-incubated with RGD peptide followed by exogenous application of Shv-GFP protein. (I) Representative images of cell spreading and phospho-FAK levels in S2 cell treated with the indicated media. (J) Quantification of the relative phospho-FAK levels in S2 cells conditioned with the indicated media. All values represent mean ± SEM and n is indicated in the bar graph. For multiple samples, One-way ANOVA followed by post hoc analysis with Bonferroni’s multiple-comparison test was used to determine statistical significance. * p < 0.05 compared to control. All scale bars = 5 μm.

Having established that Shv genetically interacts with integrin to maintain stem cell niche integrity, we tested whether Shv activates integrin signaling. Based on the presence of a signal peptide sequence, we propose Shv is secreted to activate integrin pathway extracellularly. To examine secretion of Shv, we transfected *Drosophila* Schneider’s (S2) cells with Shv tagged with V5 and assayed for the presence of Shv in the media. Shv was detected in the media of transfected cells, but not if the signal peptide was removed from Shv (NoSP-Shv; Fig 4A), indicating Shv is a secreted protein. To understand whether Shv activates integrin via an outside-in signaling mechanism, we tested the ability of extracellularly applied Shv to activate integrin by measuring the extent of focal adhesion kinase (FAK) phosphorylation. Previous studies have indicated a strong correlation between integrin activation and autophosphorylation of FAK at Tyr397 site [34,62,63]. Fig 4B shows that addition of Shv containing media to untransfected S2 cells was sufficient to increase pFAK levels and induce cell spreading that is indicative of cell adhesion activation as detected by phalloidin staining. However, treatment of cells with media collected from cells transfected with NoSP-Shv failed to induce any change in cell shape or pFAK levels. Preabsorption of Shv (Shv pull down) from the media also prevented both effects, indicating extracellular Shv is responsible for the changes (Figs 4B, 4C and S6A). Furthermore, reducing βPS integrin levels using *mys-RNAi* abolished the effects of Shv on cell spreading and FAK phosphorylation (Figs 4D, 4E and S6B), suggesting that extracellular Shv induces cell spreading through integrin activation. To rule out the possibility that Shv modulates integrin signaling via an inside-out manner, we examined the levels of FAK phosphorylation in mock, Shv, and NoSP-Shv expressing cells incubated with fresh media. Despite the presence of intracellular Shv, no change in cell shape or FAK phosphorylation was detected when Shv was removed from the media (Fig 4F and S6C Fig). Together, these data support the claim that extracellular Shv is both necessary and sufficient for integrin activation.

We next examined whether Shv colocalizes with βPS integrin receptors by generating Shv with GFP tagged at the C-terminus. We found Shv-GFP is secreted into the media and can activate integrin signaling as indicated by increased FAK phosphorylation (S6D and S6E Fig). Extracellularly applied Shv-GFP also colocalized with βPS integrin receptors on the S2 cell surface (Fig 4G). Furthermore, we found that pre-incubation of S2 cells with the RGD peptide competed against the ability of Shv-GFP to adhere to the S2 cell surface (Fig 4H and S6F Fig), suggesting that Shv interacts and binds to integrin receptors. Integrin receptors have been shown to bind to a wide variety of ligands, including, but not exclusively, to those that contain the RGD sequence [64]. Sequence alignment shows that even though the RGD sequence in human DNAJB11 is not conserved in fly, KND sequence in Shv does contain sequence similarity to RGD (highlighted in S2A Fig). Furthermore, a similar sequence, KGD, had previously been shown to be sufficient for integrin activation [65]. To test whether KND sequence belonging to Shv is required for activation of integrin, we mutated the charged residues of KND to hydrophobic amino acids, LNV (Shv^LNV^). While Shv^LNV^ is still secreted by S2 cells (S6G Fig), it diminished the ability of Shv to activate integrin signaling as depicted by the absence of cell spreading and comparable pFAK levels to control (Fig 4I and 4J). Together, our results suggest that Shv can bind to and interact with integrin directly to modulate integrin activation.

### Secretion of Shriveled activates integrin signaling and modulates E-cadherin levels *in vivo*

**Fig 5:**
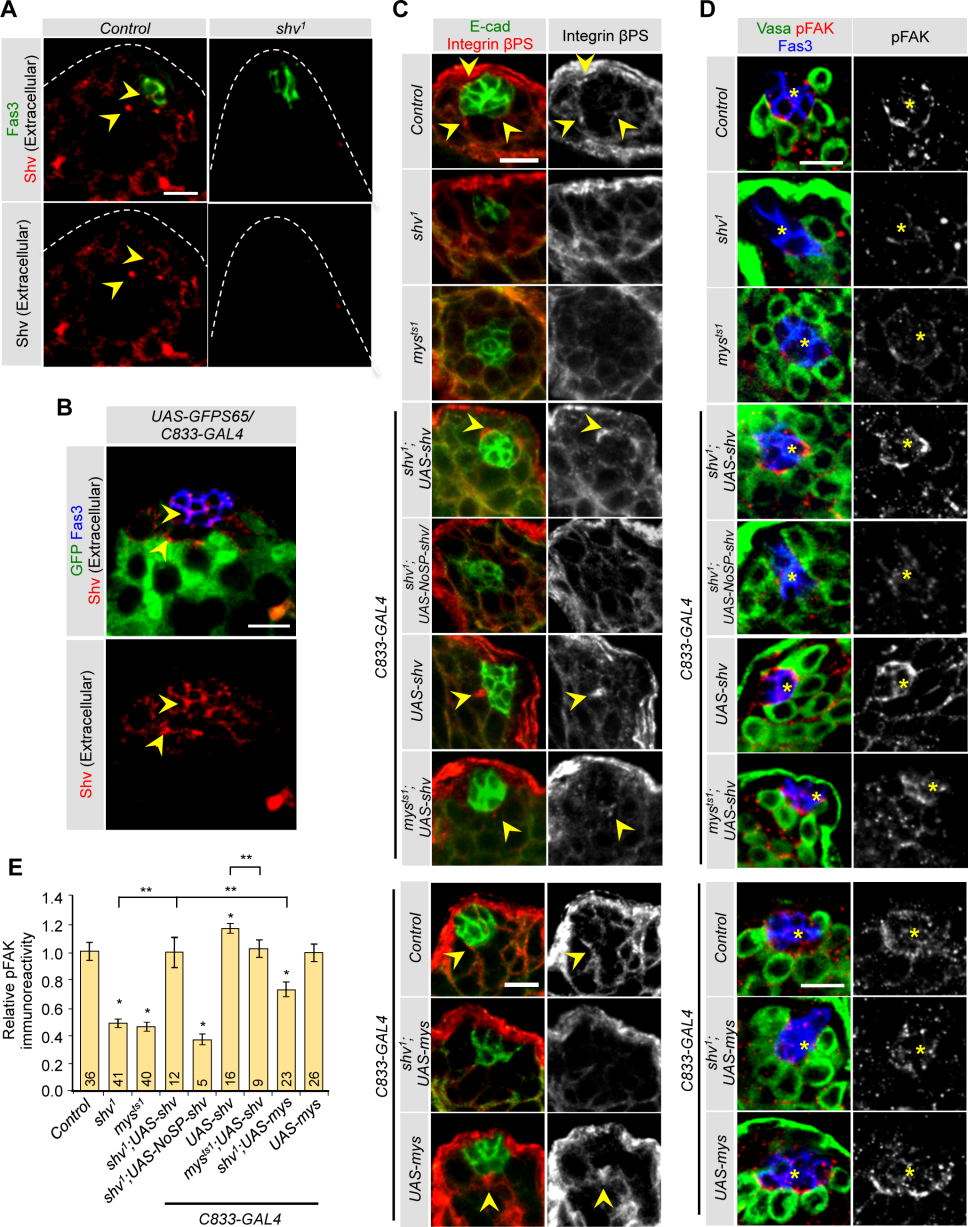
Shv activates βPS integrin signaling *in vivo*. (A) Staining of Shv antibody without detergent shows extracellular Shv accumulation (arrowhead), whereas punctate extracellular Shv is absent in *shv*^*1*^. (B) Arrows point to extracellular Shv labeling in hub and GSC/CySC interface. (C) Integrin clustering revealed by staining with βPS integrin antibody staining (yellow arrowheads). *shv*^*1*^ mutant show localized decrease in integrin clustering. (D) Levels of FAK phosphorylation (pFAK) at the hub/CySCs interface in the indicated genotypes. Yellow asterisks highlight the hub. (E) Quantification of the relative pFAK intensity normalized to Fas III levels across genotypes. * p < 0.05 compared to control and ** p < 0.05 compared to indicated genotypes. All values represent mean ± SEM and n is indicated in the bar graph. Age of flies examined is 3 days after eclosion. For multiple samples, One-way ANOVA followed by post hoc analysis with Bonferroni’s multiple-comparison test was used to determine statistical significance. All scale bars = 10 μm.

Having demonstrated that Shv is a secreted protein that activates integrin signaling *in vitro*, we next tested if Shv is present extracellularly in fly testes. Shv antibody staining of the fly testes without detergent confirmed the extracellular presence of Shv (Fig 5A and 5B). Shv signal was observed at the tip region, present as puncta at the hub and GSC/CySC interface regions of the control testes but absent in *shv*^*1*^. Next, we determined if Shv can indeed activate integrin signaling in stem cell niche *in vivo* by examining 1) integrin clustering, and 2) FAK phosphorylation, both of which strongly correlate with integrin activation [34,62,63]. Testes from control adult flies showed strong integrin staining at the hub/CySC interface (Fig 5C), consistent with previous reports [13,38,57]. Strong phosphorylated FAK (pFAK) signals were also seen at the hub/CySC interface (Fig 5D). *mys*^*ts1*^ mutants showed reduced integrin and pFAK levels, confirming pFAK is indeed sensitive to integrin signaling (Fig 5D and 5E). *shv*^*1*^ mutants showed a virtual absence of integrin clustering and reduced pFAK staining at the hub/CySC border that is markedly enhanced by upregulation of full-length Shv in mutant background. To further confirm that secretion of Shv is necessary for integrin activation *in vivo*, we generated transgenic flies containing NoSP-Shv under UAS control. Expression of NoSP-Shv in *shv*^*1*^ mutant background did not restore integrin clustering and pFAK levels (Fig 5C–5E), supporting the claim that integrin activation and clustering is achieved by extracellular Shv. Furthermore, upregulation of Shv alone was sufficient to increase integrin clustering and pFAK staining at the hub/CySC border, but these were dampened in *mys*^*ts1*^ mutant background. This result is consistent with Shv being an activator of integrin *in vivo*. Interestingly, elevation in localized integrin signaling at the hub/CySC border in *shv* overexpression testes is accompanied by a reduced number of GSCs (S5A Fig), consistent with previously reported integrin-dependent niche competition between GSCs and CySCs [13].

*shv*^*1*^ mutants also showed a dramatically reduced DE-cadherin level in the hub cells. One plausible mechanism is that Shv regulates DE-cadherin level through integrin. We first tested this hypothesis *in vitro* using S2 cells. Incubation of S2 cells with Shv containing media elevated DE-cadherin signal compared to cells treated with control media. Knockdown of βPS integrin using *mys-RNAi* blocked the effects of Shv in triggering DE-cadherin elevation (S7A and S7B Fig). These results not only suggest that Shv can induce DE-cadherin expression but confirm that Shv regulates DE-cadherin level through integrin activation.

**Fig 6:**
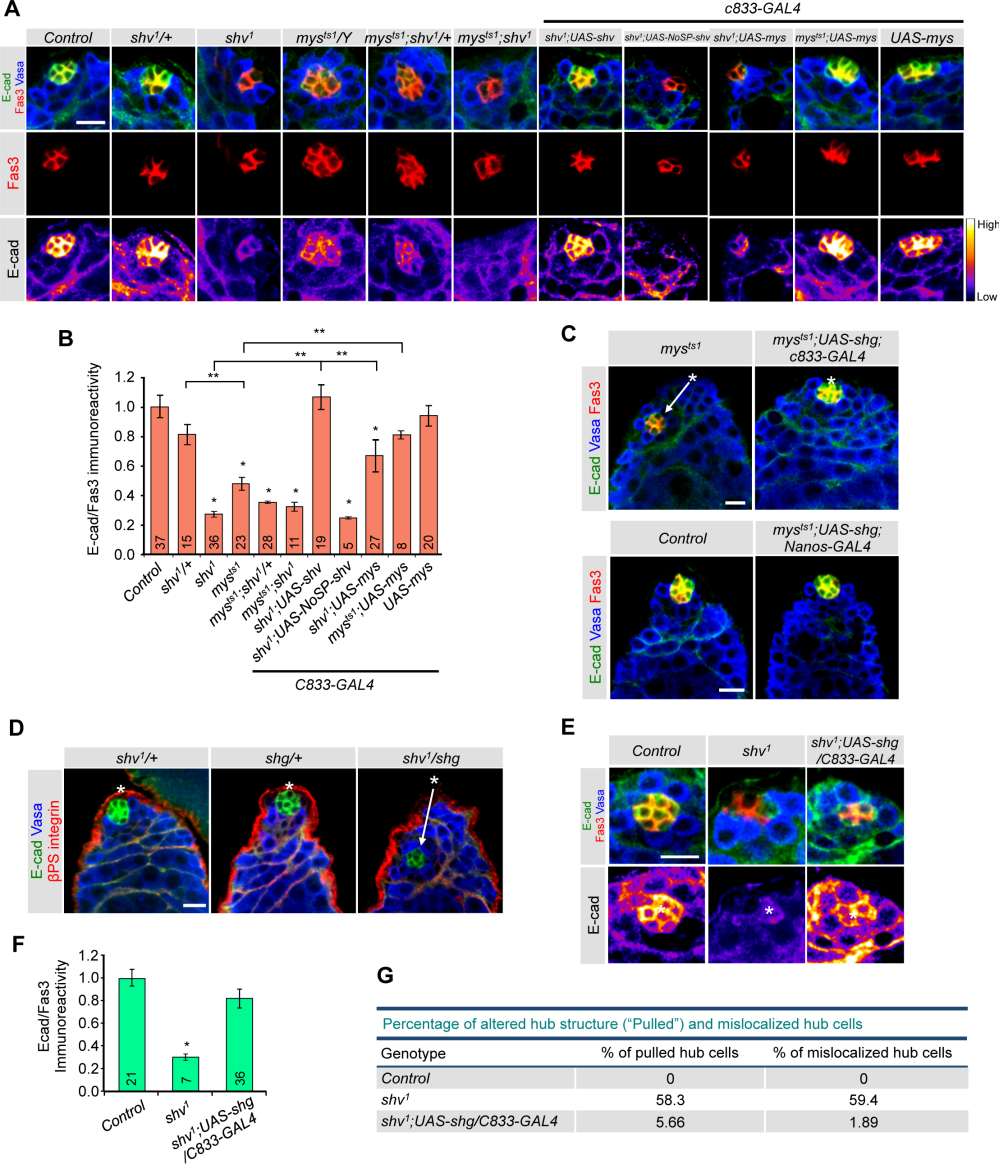
Shv regulates DE-cadherin level through βPS integrin. (A) Images of the apical tip of testes stained with DE-cadherin (E-cad) and indicated antibodies. Lower panels show pseudo-colored images of DE-cadherin staining intensity. (B) Quantification of the relative DE-cadherin intensity normalized to Fas III levels across genotypes. (C) Upregulation of DE-cadherin in *mys*^*ts1*^ background rescued hub mislocalization. (D) Reducing Shv and DE-cadherin (shg) caused hub mislocalization. Asterisks highlight the apical tip of the testes and arrows point the observed hub position. (E) Images of testes stained with DE-cadherin and indicated antibodies. (F) Quantification of the relative DE-cadherin intensity normalized to Fas III levels across genotypes. (G) Table showing percentage of altered hub structure and mislocalized hub cells. * p < 0.05 compared to control and ** p < 0.05 compared to indicated genotypes. All values represent mean ± SEM and n is indicated in the bar graph. Age of flies examined is 3 days after eclosion. For multiple samples, One-way ANOVA followed by post hoc analysis with Bonferroni’s multiple-comparison test was used to determine statistical significance. All Scale bars = 10 μm.

Next, we took a genetic approach to test *in vivo* that DE-cadherin expression can indeed be regulated by integrin activation. We focused on DE-cadherin levels since it has previously been shown to be essential for hub compaction, GSC and CySC maintenance [28,31]. We found that reducing βPS integrin in the testes led to lower DE-cadherin staining in the hub cells that is restored by expressing *mys* in *mys*^*ts1*^ using *c833-GAL4* driver (Fig 6A). To further confirm that this decrease in DE-cadherin intensity is specific and not due to a change in the number of hub cells, we plotted the relative DE-cadherin intensity normalized to Fas III intensity (Fig 6B). Again, *mys*^*ts1*^ showed reduced level of DE-cadherin to Fas III ratio, consistent with the claim that integrin regulates DE-cadherin expression in the hub cells. Expression of full-length, but not NoSP-Shv, rescued the *shv*^*1*^ mutant phenotype, again verifying that secretion of Shv is important for normal DE-cadherin level. Expression of NoSP-Shv in *shv*^*1*^ using the *nanos-GAL4* driver also resulted in the similar findings for DE-cadherin expression (S7C and S7D Fig). Furthermore, double mutants *mys*^*ts1*^*;shv*^*1*^ showed a similar decrease in DE-cadherin level as single mutant (Fig 6A and 6B), supporting the notion that Mys and Shv is in the same pathway to regulate DE-cadherin level. We found that overexpression of *mys* in *shv*^*1*^ partially rescued the decrease in DE-cadherin level (Fig 6A and 6B). This weak rescue is consistent with our findings that Shv needs to be present for efficient integrin activation and thus DE-cadherin expression.

Because our data indicates that integrin alters DE-cadherin levels in hub cells, we next tested whether this reduction in DE-cadherin level contributes to hub mislocalization. Fig 6C shows that upregulating DE-cadherin in *mys*^*ts1*^ mutants sufficiently restored hub location. Furthermore, while heterozygous mutants of *shv*^1^ (*shv*^*1*^*/+*) and DE-cadherin (*shotgun* in *Drosophila*; *shg/+*) did not show hub mislocalization, *shv*^*1*^*/shg* mutants have hub cells distantly located away from the tip (Fig 6D and S7E Fig; 85% of testes examined). Previous reports demonstrated that loss of DE-cadherin alone is not sufficient to cause hub mislocalization [26]; however, our results imply that although the primary role of DE-cadherin in hub cells is not to anchor the hub, it does contribute to hub localization.

We next asked if reduction in DE-cadherin level contributes to the *shv*^*1*^ mutant phenotypes. To this end, we restored DE-cadherin level in *shv*^*1*^ using the *c833-GAL4* driver (Fig 6E and 6F). We found that elevating DE-cadherin in *shv*^*1*^ significantly rescued the hub “pulling” and hub mislocalization phenotype (Fig 6E, 6F and 6G). Moreover, GSC loss of *shv*^*1*^ was prevented by DE-cadherin upregulation (average GSC number in *shv*^*1*^*;UAS-shg-GFP/c833-GAL4* is 8.45 ± 0.23 vs. 6.27 ± 0.58 in *shv*^*1*^, p < 0.05). However, it did not rescue the number of hub cells of *shv*^*1*^ to control levels, suggesting that restoring DE-cadherin alone is not sufficient, presumably because activation of integrin signaling by Shv affects multiple downstream pathways/targets in addition to DE-cadherin, such as N-cadherin.

### Upregulation of Shv preserves stem cell niche during aging

**Fig 7:**
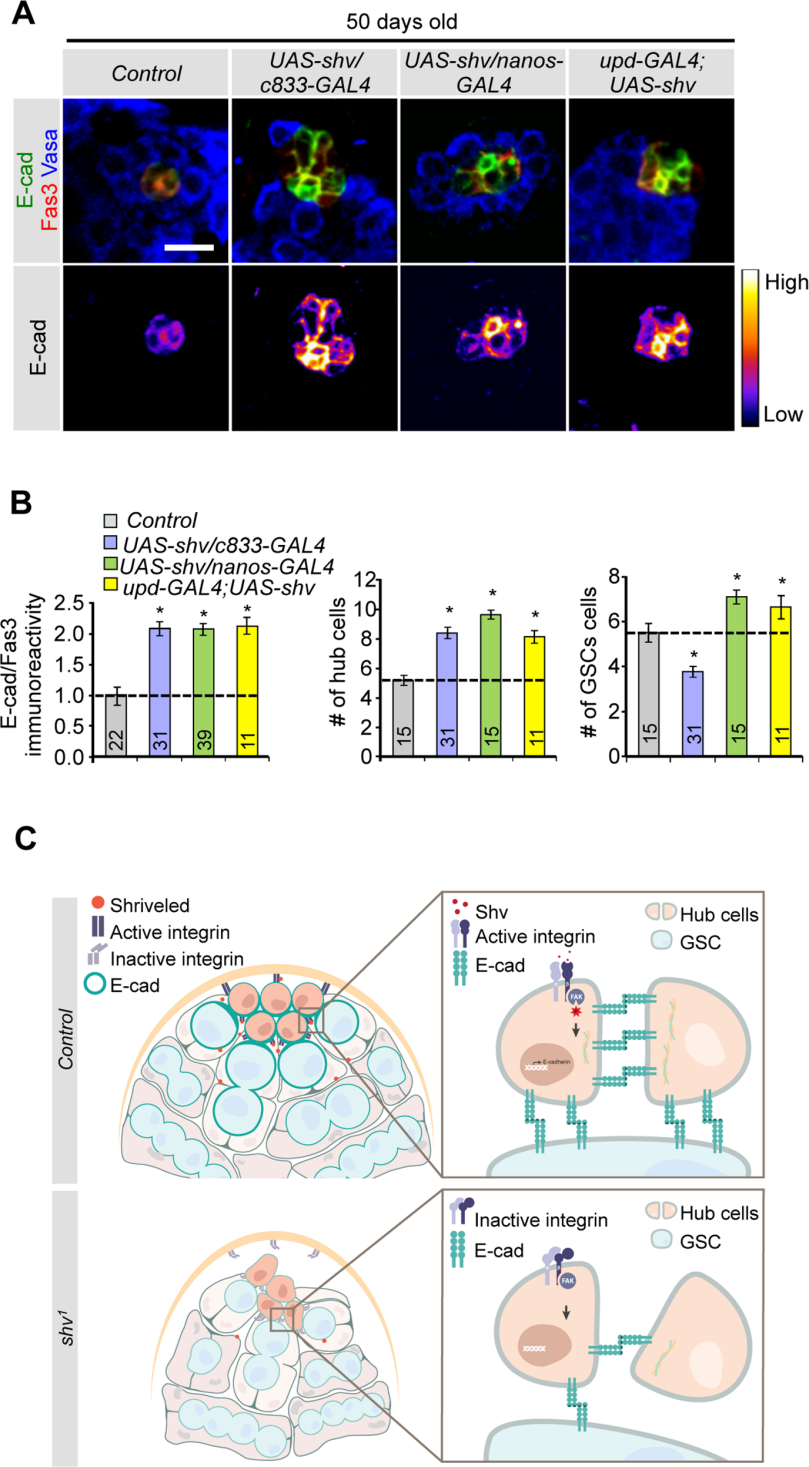
Upregulation of Shv prevents loss of niche and GSCs during aging. (A) Staining of testes dissected from 50 days old males for the indicated genotypes. Upregulation of Shv preserved DE-cadherin intensity during aging. Scale bar = 10 μm. (B) Quantification of DE-cadherin intensity, average hub cell number per testis, and average GSC number per testis in 50 days old flies. Sample numbers are indicated in the graph. For multiple samples, One-way ANOVA followed by post hoc analysis with Bonferroni’s multiple-comparison test was used to determine statistical significance. All values represent mean ± SEM and * p < 0.05 compared to control. (C) Model for how Shv maintains niche integrity in the *Drosophila* testes. In control testis (top), Shv activates integrin receptors, promoting hub anchoring at the tip. Shv-dependent integrin activation also increases DE-cadherin expression, thus enhancing hub-hub and hub-GSC cell adhesion to maintain hub architecture, niche integrity, and GSC health. In the absence of Shv (*shv*^*1*^ mutant; bottom), loss of integrin activation causes the hub cells to drift away from the tip, and leads to disrupted cell-cell adhesion. This results in altered niche structure, reduced number of GSCs, and eventually loss of the hub as observed in *shv*^*1*^ mutant.

Having demonstrated that loss of Shv results in deterioration of hub architecture and stem cell niche integrity, we next tested whether upregulation of Shv could prevent decline in hub cell number and DE-cadherin expression in older animals. Interestingly, upregulation of Shv in hub cells, hub+cyst cells, or GSCs preserved hub cell number and DE-cadherin levels in 50 days old testes when compared to age matched control testes. Only expression of Shv in hub cells or GSCs (but not hub+cyst cells using *c833-GAL4*) preserved the number of GSCs during aging (Fig 7A and 7B). Again, these results are consistent with integrin-dependent competition for niche space by somatic cells [13]. We also determined if Shv level in the testes changes with age. Immunostaining of testes dissected from young and older flies show that Shv level is reduced in 30 days old flies (S7F Fig). This observation is consistent with our model that Shv is required for the maintenance of hub structure since testes from older flies are known to have reduced DE-cadherin levels and reduced number of hub cells [12]. Together our results indicate that upregulation of Shv has the ability to preserve stem cell niche during aging.

## Discussion

In this study, we identify Shriveled as a key factor in preserving niche integrity and GSC number in the *Drosophila* testes *in vivo*. We show that Shv is a secreted protein that activates integrin signaling and in turn controls DE-cadherin levels. While previous experiments suggest that integrin and DE-cadherin work independently in steps involving hub anchoring and GSC attachment to the hub [19,26,28,29,37], respectively, our results reveal a tight link between integrin activation and DE-cadherin levels in hub cells. Cooperation between integrin and DE-cadherin signaling may serve to ensure maintenance of the 3-dimensional structure of the hub by promoting hub-hub cell interaction and GSC-hub adhesion following hub anchoring. Furthermore, secretion of Shv by somatic cells and GSCs may act as a feedback signal to sustain optimum integrin activation, DE-cadherin expression, and a healthy stem cell niche during aging (modeled in Fig 7C).

Our immunostaining data revealed that Shv is found in multiple cell types and in distinct subcellular locations in the *Drosophila* testes. Due to the striking hub deterioration phenotype seen in *shv*^*1*^ mutants, we focused our studies at the apical tip. We found that despite the presence of *shv* RNA in multiple cell types including the GSCs, Shv protein was not detected inside the GSCs but often seen at the hub/GSCs or germ/cyst cell borders. Together with our *in vitro* data showing release of Shv extracellularly, we conclude that Shv is secreted efficiently in the testes by GSCs. Consistent with this interpretation, expressing Shv in GSCs rescued the *shv*^*1*^ phenotype, but not if the signal peptide had been deleted. Hub and CySCs also likely secrete Shv, as it is also present at hub-hub or hub-CySC cell interface and expression of Shv using hub+cyst cell driver rescued the mutant phenotype. Together, extracellular Shv may allow multidirectional communication between different cell types for niche maintenance. Note that based on reports showing hub mislocalization does not cause sterility [26,27], we do not think that altered hub structure and localization seen in *shv*^*1*^ directly contributed to sterility in young flies. Given that Shv is also found in the nucleus spermatocytes, it is likely that Shv plays multiple roles during spermatogenesis. Future studies examining Shv function in the nucleus and factors regulating Shv subcellular distribution will lead to better understanding of its multiple roles in *Drosophila* testes and spermatogenesis.

Upon release, Shv activates integrin signaling. This is supported by 1) genetic data demonstrating interaction between Shv and βPS integrin, and 2) *in vitro* data showing extracellular Shv application triggers integrin-dependent changes in pFAK, and 3) mutating Shv at potential integrin-interaction sites diminished its ability to activate integrin signaling. Based on our *in vitro* data that pre-incubation of RGD peptide can block the ability of Shv to bind to integrin, we believe that Shv can bind to Position Specific 2 (PSs) integrin group, which mediates binding through RGD peptide. In *Drosophila*, it is thought that αPS3, αPS4, αPS5 and one βPS subunit (*mys*) are expressed in the gonads [26,66]. It is thus possible that a single cell expresses multiple integrin receptors comprised of different αPS subunit together with βPS, thus allowing it to bind to different ligands to promote adhesion in a temporal and spatial manner. Furthermore, integrin receptors have different affinity for different ligands. We thus envision that Shv acts to serve a modulatory role, and not to out compete the binding of normal ligand. Altogether, our results demonstrate that Shv is a novel ligand that interacts and activates integrin extracellularly.

Our finding that Shv-dependent integrin signaling modulates DE-cadherin expression in hub cells is surprising, since these two adhesion molecules are thought to act in separate pathways involving hub anchoring and GSC attachment. Nevertheless, the effects of integrin activation on DE-cadherin levels in the hub cells were not examined previously, and while reducing DE-cadherin levels alone did not alter hub positioning [26], contribution of DE-cadherin to hub anchoring could not be ruled out. Indeed, we showed that DE-cadherin contributes to hub anchoring, apparent only when integrin function is compromised. Furthermore, DE-cadherin expression was increased upon extracellular application of Shv, but not if integrin receptors were silenced, revealing direct relationship between DE-cadherin and integrin signaling pathway. Shv-dependent integrin activation may lead to transcriptional and/or translational increase of DE-cadherin rather than re-localization of DE-cadherin to the cell surface. This is supported by observations in S2 cells showing an overall elevation of DE-cadherin intensity rather than membrane redistribution following Shv incubation. In addition, consistent with data that loss of DE-cadherin disrupts hub compaction and reducing DE-cadherin in hub and CySCs leads to hub cell loss [28,31], *shv*^*1*^ mutants showed a hub compaction phenotype in which the hub cells appear pulled away from the apical tip and a gradual loss of hub cells during aging. Indeed, upregulation of DE-cadherin in *shv*^*1*^ testes successfully restored hub mislocalization as well as pulled hub structure, supporting the claim that DE-cadherin serves downstream of Shv to maintain healthy stem cell niche structures. Taken together, it is likely that Shv-dependent integrin activation enhances cadherin expressions, which ensures proper cell-cell adhesion during aging. This may result in a physical barrier that passively contributes to hub cell anchoring by decreasing the ease by which hub cells could move away from the tip. Note that although integrin activation modulates cadherin expression, other intrinsic and extrinsic factors likely regulate cadherin expression independently, especially during early embryogenesis. This may explain why *mys* null mutants still showed normal GSCs formation around the mis-positioned hub cells during embryonic stage [26]. However, severe reduction in integrin signaling in adults did lead to an age-dependent loss of hub [26], similar to what was observed in *shv*^*1*^ mutants.

The identification of a novel activator of integrin signaling may have broad implications since integrin is involved in a wide range of biological processes ranging from development to cancer [67]. Interestingly, the human homolog of Shv, DNAJB11, has recently been identified in secretome profiling as a protein upregulated in oral cavity squamous cell carcinoma [68], as well as secreted during unfold protein response activation [52]. In addition, DNAJB11 has been shown to be secreted in mice and influences integrin signaling [51,69], suggesting that the functions of Shv may be evolutionarily conserved. Strikingly, we found that upregulation of Shv was sufficient to preserve the number of hub cells and GSCs that normally decline during aging. An understanding of how Shv functions in niche maintenance may thus contribute to future stem cell therapy, as activation of integrin by Shv could potentially enhance our ability to optimize niches and promote survival of *in vitro* derived stem cells after transplantation.

## Materials and Methods

### Fly stocks and antibody generation

Flies were cultured at 25°C on standard cornmeal, yeast, sugar, and agar medium unless indicated otherwise. White-eyed flies (*w*^*1118*^) were used as wildtype throughout all experiments. The following fly lines were used with Bloomington Stock # in parenthesis: *mys*^*ts1*^*(# 3169)*, *c833-gal4 (# 6988)*, *nanos-gal4 (#4937)*, *upd-gal4* (from D. Harrison), *c587-gal4*, *shg (#26885)*, *shv*^*9803*^ (# 9803), *shv*^*C00496*^ (from Exelixis at Harvard Medical School), *UAS-Shg-GFP (#58445)*, *UAS- DEFL(II)* (from Y. Yamashita), *c587-gal4* (from E. Matunis and A. Spradling), *UAS-MYS* (from R. Xi). *shv*^*1*^ mutant was generated using random hop with *Drosophila* lines carrying p{lacZ,w^+^} [45]. The site of P element insertion was determined by plasmid rescue [70]. *shv*^*PJ*^ was generated by precise excision of the P-element allele. Full length s*hv* transgene construct was generated by subcloning the coding regions of *CG4164* into the pINDY6 vector, and Shv with signal peptide deleted (NoSp-shv) was generated by deleting the first 22 amino acids corresponding to the predicted signal peptide as determined using SignalP 4.1. Transgenic flies were generated by standard transformation method [71]. Affinity purified rabbit polyclonal antibody for Shv was generated against amino acids 243–256 of Shv (Sigma Genosys). All other stocks and standard balancers were obtained from Bloomington Stock Center (Bloomington, IN).

### Immunocytochemistry

Testes were dissected in PBS and fixed in 4% paraformaldehyde for 25 min. Fixed samples were washed with 0.1% triton X-100 in PBS (PBST) then blocked with 5% normal goat serum (NGS) in PBST. Primary antibodies were diluted in blocking solution and used as following: rabbit anti-Shv, 1:400; rat anti-Vasa, 1:20 (DSHB); rabbit anti-Vasa 1:5000 (gift from P. Lasko); mouse anti-Fasciclin III, 1:15 (7G10, DSHB); anti-adducin, 1:35 (1B1, DSHB); rat anti-DE-cadherin, 1:20 (DSHB); mouse anti-integrin βPS, 1:100 (DSHB); rat anti-DN-cadherin, 1:10 (DSHB); rabbit anti-phosphoFAK, 1:200 (Invitrogen). Secondary antibodies used were Alexa-488, 555 or 405 conjugated, 1:250 (Invitrogen). Extracellular labeling of Shv protein was adapted from Zheng et al. [72]. Briefly, testes were dissected in cold Ringer’s solution, incubated with anti-Shv antibody in cold Ringer’s solution containing 5% NGS for 2 to 3 hrs at 4°C. Testes were then washed 3 times with cold Ringer’s solution and processed for standard immunostaining. Images were captured using Zeiss LSM5 confocal microscope using a 63X 1.6NA oil immersion objective with a 1x or 2x zoom. When comparing intensity across genotypes, the exposure time was kept constant for all genotypes per experiment. The staining intensities of pFAK and DE-cadherin were normalized to Fasciclin III staining that labels the hub cells. All values were normalized to control done within the same experimental set.

### Antibody preabsorption for Western and immunocytochemistry

Antibody-peptide absorption was achieved by incubating 0.2ug of Shv peptide with 1 μg of Shv antibody for 1hr at RT. Western blots and standard immunocytochemistry were carried out as described earlier.

### Cell counting and hub positioning determination

The number of hub cells was determined by counting DAPI nuclei that were positive for Fasciclin III marker. Germ cells contacting the hub that were adducin and Vasa positive were counted as GSCs. Image J was used to measure the DE-cadherin signal intensity as defined by Fasciclin III positive hub area. DE-cadherin level was normalized to the hub area. Hub location was determined by measuring the distance from the apical tip of the testes to the center of the hub area. In addition, we strictly scored the hub as mislocalized when it is located more than two Vasa positive germ cells apart, or 14 μm, from the apical tip of the testes. We scored the hub as “pulled”, or with altered structure, when a cross-section image of testis stained with Fascicilin III is not circular.

### Fluorescence in situ hybridization

*shv* RNA detection was performed following protocol by Toledano et al., except testes were incubated with PBS+0.1% triton for five minutes following fixation and prior to primary antibody addition [56]. Probes for shv was generated by PCR using primers against *shv* (F: ATGCAGCTTATCAAGTGCTT and R: TCACAGTCCATTGTATATGC) and subcloned into pGEM-Teasy (Promega)

### Western blotting

To detect Shv levels in flies, protein extracts were obtained by homogenizing flies in RIPA lysis buffer (50 mM Tris-HCl, pH7.5, 1% NP-40, 0.5% NaDoc, 150 mM NaCl, 0.1% SDS, 2 mM EDTA, 50 mM NaF, 1 mM Na_3_VO_4_, 250 nM cycloporin A, protease inhibitor cocktail (Roche) and phosphatase inhibitor cocktail 1 (Sigma) using mortar and pestle. 20 μg protein homogenate was separated by SDS-PAGE and transferred to nitrocellulose membranes. For Western blots using S2 cell extracts, 2 μg of cell protein extracts or 15 μl of media were loaded. Primary antibodies were diluted in blocking solution as following: rabbit Shv, 1:500; anti-tubulin 1: 500 (7E10, DSHB); anti-βPS integrin, 1:1500 (from R. Hynes); anti-V5, 1:5000 (Invitrogen).

### Quantitative RT-PCR

One μg of total RNA from adult testes was isolated using TRIzol reagent (Invitrogen), converted to cDNA using SuperScriptII reverse transcriptase (Invitrogen), and then used for quantitative RT-PCR with SYBR Green reagent (Applied Biosystems). Primers were designed from mRNA sequence to detect *shv* and normalized *gapdh* transcripts (shv: F- CCATGGAGATCAAGCACCTT, R-TTTCTTGAGCGCTTCCTTGT; GAPDH: F-TGGTACGACAACGAGTTTGGC, R-GTCTCACCCCATTCTACCGC; *crq*: F- TTCTCATCACCGGCATCACG, R-GCTATCACAAACTGCAAGACG). Thermocycling was conducted in The Light Cycler 480 Real-Time PCR system (Roche). The Light Cycler Analysis Software 4.05. (Roche) was used to analyze amplification plots. The relative quantity of amplified cDNA corresponding to each gene was calculated by using the ΔΔ*C*t method and normalized for expression of *gapdh* in each sample.

### S2 cell culture

*Drosophila* S2 cells (Invitrogen) were cultured at 29°C in Schneider’s insect medium (Sigma-Aldrich) supplemented with 10% FBS. Full length Shv and Shv with signal peptide deleted (noSP-Shv) were subcloned into pAc5.1/V5-His vector (Invitrogen). Shv-GFP is generated by inserting eGFP sequence to c-terminus of full-length Shv and subcloned into the same pAc5.1/V5-His vector. Transfection was performed using Calcium Phosphate Transfection Kit (Invitrogen). To test for secretion of Shv, transfected cells and media were harvested 72 hours after transfection. For cell spreading assay and pFAK determination, untransfected S2 cells were seeded in 24-well plate containing 12mm glass coverslip pre-coated 50 μg/ml of poly-D-lysine (Millipore) at 0.5X10^6^cells/well. Filtered media (0.2μm polyethersulfone membrane, VWR) collected from transfected cells were added to each well, and cells were allowed to spread for 1hr at 29°C. Amount of media applied was kept at 1:2 ratios to the seeded volume. Following aspiration, cells were fixed and processed for staining as described above. To remove Shv from the collected media (Shv pull down), collected media were incubated with anti-V5-agarose beads (Sigma Adrich) at 4° for 2hr. For control, collected media was incubated with agarose A/G beads (Santa Cruz Biotechnology) for the same amount of time. Beads were washed with 1X PBS four times. Pull-down was confirmed via western blots using antibody against V5. For colocalization experiment, detergent was omitted in all solutions. Primary antibodies were used as following: rabbit anti-phosphoFAK, 1:400 (Invitrogen); rabbit anti-GFP, 1:1000 (Acris); mouse anti-V5, 1:5000 (Invitrogen); rat anti-DCAD2 1:20 (DSHB); mouse anti-integrin βPS, 1:100 (DSHB). 100uM working stock of Actin-stain 488 fluorescent phalloidin (Cytoskeleton) was used to stain actin filaments. Coverslips were mounted in Pro-long Gold Antifade reagent with DAPI (Invitrogen). For RGD peptide treatment, 1mg/mL of RGD peptide (Sigma Aldrich, A8052) was added for 15 min prior to application of Shv containing media.

### dsRNA construction

cDNA was synthesized from S2 cell RNA using TRIzol reagent followed by SuperScriptII reverse transcriptase under the recommended manufacturer’s conditions. 511 bp fragments of *myospheroid* (*mys*) gene were amplified by PCR with Taq polymerase (KapaBiosystem). Double-stranded RNA (dsRNA) primer sequences were obtained from *Drosophila RNAi Screening Center* (DRSC) #18799 and tailed with T7 sequences (F: CCTCTTCGGTGGAGATGAA, R: GGATTTGGTCGCTTGTGG). dsRNA was synthesized using MEGAscript T7 Kit (Ambion) according to the manufacturer’s instructions. 2μg dsRNA was introduced in S2 cells using Effectene Transfection Reagent (Qiagen) and cells were harvested after 72hrs.

### Site-directed mutagenesis

The KND sequence on Shv was mutated to LNV (Shv^LNV^) using QuickChange Kit (Stratagene). Oligonucleotide primers used for site-directed mutagenesis were (with mutated nucleotides underlined: F- CATCCGCGATTCCTGCGCCTGAATGTTGAT CTGTACACGAACGT and R-ACGTTCGTGTACAGATCAACATTCAGGCGCAGGAAT CGCGGATG. Point mutation was confirmed by sequencing (Genewiz).

### 3D reconstruction

Confocal z-stack images were volume rendered for 3D reconstruction using Imaris 7.7 software (Bitplane). Additional surface rendering was performed toward the red channel with Imaris 7.7 software.

### Statistics

For paired samples, Student’s T-test was used. For multiple samples, One-way ANOVA followed by post hoc analysis with Bonferroni’s multiple-comparison test was used to determine statistical significance. All plots show mean ± SEM.

## Supporting Information

S1 Fig Hub cell and CySC quantification in different ages and altered hub architecture seen in *shv*^*1*^ mutants. (A) Quantification of control and *shv*^*1*^ larval hub and GSC. (B) Montage showing individual confocal z-stack images of control and *shv*^*1*^ mutant testes stained with the indicated antibodies. Note that the overall hub architecture is disrupted in *shv*^*1*^ with hub cells spread throughout different z focal planes and is not clustered at the tip. (C) Table showing percentage of altered hub structure. (D) Representative images of the testes tip stained DN-cadherin antibody. (E) Characterization of hub cells using anti-cactus antibody. (F) Quantification of CySCs by counting Zfh-1(+), Eya(-) cells. Scale bar in (B), (D) and (E) = 10 μm.
(TIF)

S2 Fig Shv sequence alignment, transcript levels, and antibody specificity. (A) Diagram depicting genomic region and P-element insertions within CG4164, which we have renamed *shriveled* (*shv*). Orange boxes indicate coding region of *shv* and gray boxes show untranslated mRNA. An adjacent gene, *crq*, is oriented in the opposite direction. Green boxes indicate *crq* coding region and darker gray boxes show untranslated mRNA. Blue inverse triangle indicates P-element insertions within the 5’-UTR of *shv*. SP represents signal peptide. A predicted nuclear localization signal (NLS) is also highlighted. Sequence alignment between human DNAJB11 and Shv are shown below. Red residues highlight the RGD sequence and the similar residues in fly. (B) Quantification of the relative *shv* and *crq* transcripts in control and *shv*^*1*^. * p < 0.05 compared to control. All values represent mean ± SEM. n = 4 independent experiments. For paired samples, Student’s T-test was used. (C) Shv antibody is specific for Shv. Western blot performed using fly extract detected with Shv antibody (control) and antibody pre-absorbed with Shv peptide.
(TIF)

S3 Fig *shv* alleles. (A) Quantification of the relative *shv* transcripts. * p < 0.05 compared to control. All values represent mean ± SEM. n = 4 independent experiments. (B) Table showing percentages of testes with altered hub structure. (C) Representative images of testes dissected from 3-day old flies. Asterisks show the apical tip of the testis and arrows highlight distally located hub. (D) Testes stained with DE-cadherin (E-cad) and indicated antibodies. Lower panels show pseudo-colored images of DE-cadherin staining intensity. (E) Quantification of GSCs per testis dissected from 3-day old flies. * p < 0.05 compared to control. For multiple samples, One-way ANOVA followed by post hoc analysis with Bonferroni’s multiple-comparison test was used to determine statistical significance. Scale bars in (C) and (D) = 10 μm.
(TIF)

S4 Fig Shv distribution in testes. Dual fluorescent *shv* RNA and protein detection to mark different cell types. (A) Control and *shv*^*1*^ testes taken at lower magnification demonstrating ubiquitously expressed *shv* RNA at the apical tip of the testis. Higher magnification images representing the presence of *shv* RNA in the spermatocytes. *shv* RNA is seen in the hub, CySCs, germ and cyst cells of the control testes, but barely detectable in *shv*^*1*^ mutant. Similarly, *shv* RNA is seen at high level in control spermatocytes and cyst cells, but not in *shv*^*1*^ mutant. (B) Control testes stained with Shv, fusome and indicated antibody to see where Shv is located. (C) Shv subcellular distribution is further investigated by staining testes expressing fluorescently-tagged organelle markers with Shv antibody. Peroxisome marker (*SKL-GFP*); lysosme marker (*SPIN-myc-eGFP);* golgi marker (*Golgi-GFP*); mitochondria marker (*mito-GFP*); ER marker (*RFP-KDEL*). For (A), (B), (C), scale bar = 10 μm. (D) Abundant Shv protein is detected in the nucleus of control spermatocytes but is significantly reduced in *shv*^*1*^ (arrowhead). Scale bar = 50 μm. Age of flies examined is 3 days after eclosion.
(TIF)

S5 Fig Restoring Shv in CySCs rescues GSC loss phenotype. (A) Quantification of the average number of GSCs per testes for the indicated genotypes. (B) Quantification of the average number of hub and GSCs per testes for the indicated genotypes. (C) Quantification of the average number of GSCs per testes for the indicated genotypes. * p < 0.05 compared to control. For multiple samples, One-way ANOVA followed by post hoc analysis with Bonferroni’s multiple-comparison test was used to determine statistical significance.
(TIF)

S6 Fig Extracellular Shv activates integrin signaling. (A) Western blot depicting Shv levels in the media for the indicated conditions. Shv pull down efficiently removed Shv proteins from the media. (B) Western blot demonstrating efficiency of *mys-RNAi*. (C) Quantification of pFAK intensity in cells transfected with the indicated constructs. Intracellular expression of Shv did not alter pFAK levels when Shv was removed extracellularly. Number of cells examined is indicated in the bar graph. (D) Representative images of cell spreading and pFAK levels in S2 cells treated with the indicated media. (E) Western blot depicting the normal secretion of Shv protein tagged with GFP. (F) Quantification of GFP levels on the S2 cell surface normalized to the amount of GFP without RGD peptide incubation. * p < 0.05 compared to control. (G) Western blot demonstrating the presence of Shv^LNV^ extracellular in the media. For paired samples, Student’s T-test was used. For multiple samples, One-way ANOVA followed by post hoc analysis with Bonferroni’s multiple-comparison test was used to determine statistical significance. Number of cells examined is indicated in the bar graph. All values represent mean ± SEM. Scale bar = 5 μm.
(TIF)

S7 Fig Secretion of Shv regulates DE-cadherin levels. (A) Representative images of cell spreading and E-cad levels in S2 and *mys-RNAi* cells treated with the indicated media. Scale bar = 5 μm. (B) Quantification of E-cad intensity in control and *mys-RNAi* transfected cells following media treatment. (C) Pseudo-colored images of DE-cadherin in 3-day old testes. Asterisks indicate the hub. (D) Quantification of relative DE-cadherin intensity across genotypes. * p < 0.05 compared to control. ** p < 0.05 between the indicated genotypes. All values represent mean ± SEM. For multiple samples, One-way ANOVA followed by post hoc analysis with Bonferroni’s multiple-comparison test was used to determine statistical significance. n is indicated in the bar graph. (E) Percentage of mislocalized hub cells observed across genotype. (F) 3 and 30 days old testes labeled with Shv and indicated antibody. Scale bar in (C) and (F) = 10 μm.
(TIF)

S1 Video 3D presentation of *Drosophila* testes stem cell niche. 3D projection rendered from confocal z-stack images of control testis stained with Vasa (green), FasIII and fusome (1B1) antibodies (red). Arrows point to the hub as visualized by Fas III staining clustered at the apical tip.
(ZIP)

S2 Video 3D presentation of *Drosophila* testes stem cell niche. 3D projection rendered from confocal z-stack images of *shv*^1^ mutant testis stained with Vasa (green), FasIII and fusome (1B1) antibodies (red). Arrows point to the “pulled” hub stained by Fas III.
(ZIP)
