# Supplementary figures and images for: Maintenance of Stem Cell Niche Integrity by a Novel Activator of Integrin Signaling
Source: PLoS Genet. 2016 May 18;12(5):e1006043. doi: 10.1371/journal.pgen.1006043 (PMC4871447; doi:10.1371/journal.pgen.1006043)

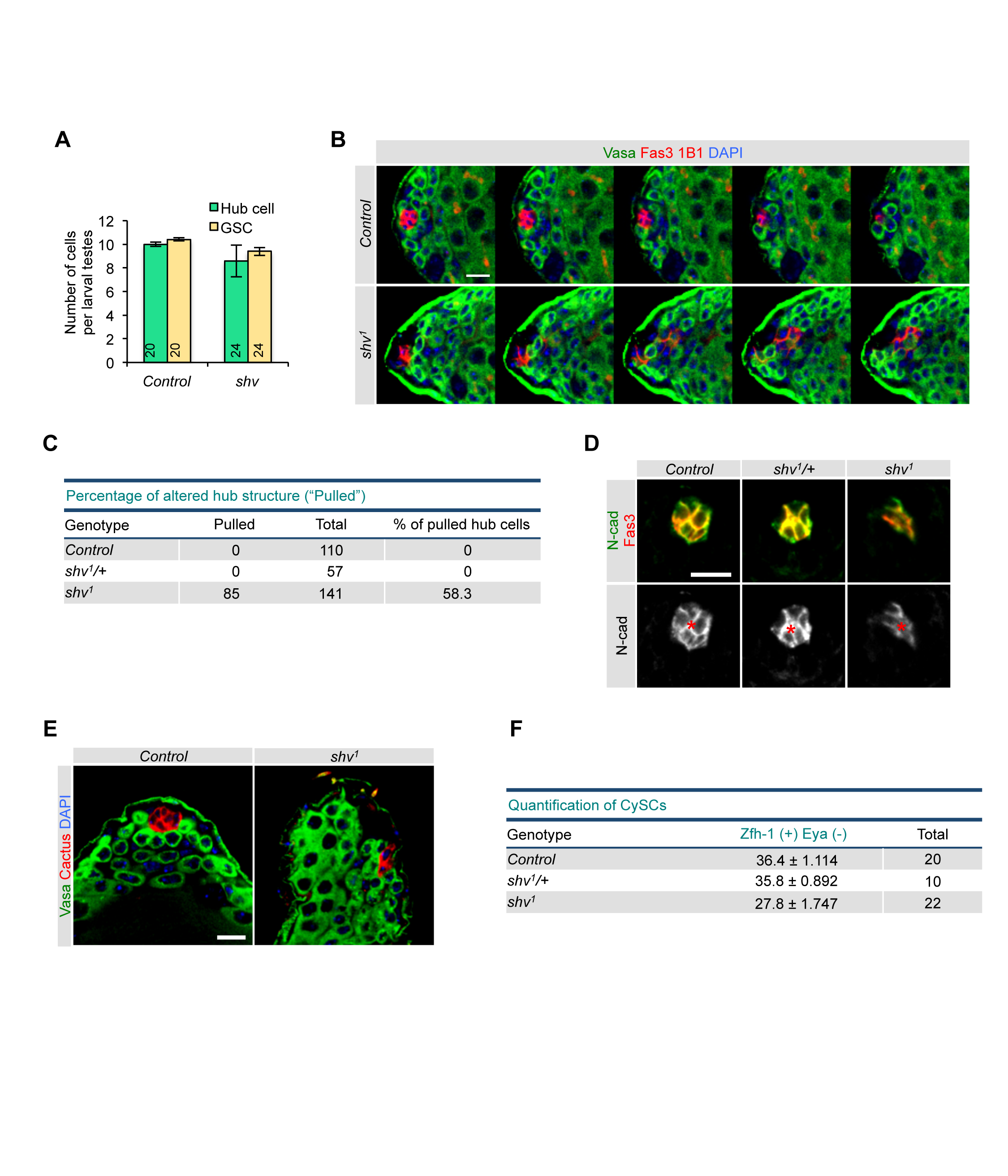

Supplement: S1 Fig — (A) Quantification of control and shv1 larval hub and GSC. (B) Montage showing individual confocal z-stack images of control and shv1 mutant testes stained with the indicated antibodies. Note that the overall hub architecture is disrupted in shv1 with hub cells spread throughout different z focal planes and is not clustered at the tip. (C) Table showing percentage of altered hub structure. (D) Representative images of the testes tip stained DN-cadherin antibody. (E) Characterization of hub cells using anti-cactus antibody. (F) Quantification of CySCs by counting Zfh-1(+), Eya(-) cells. Scale bar in (B), (D) and (E) = 10 μm. (TIF) [file pgen.1006043.s001.tif]

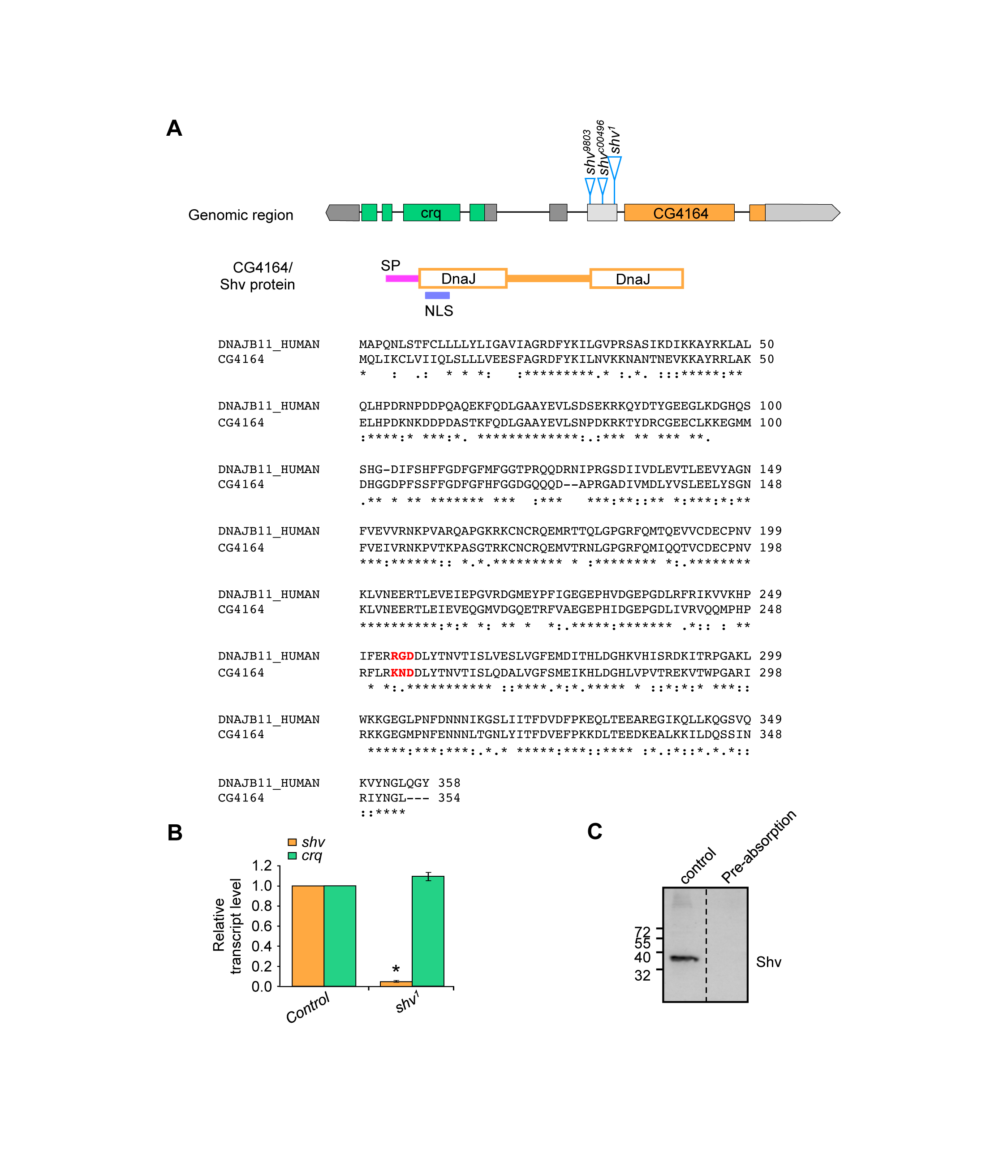

Supplement: S2 Fig — (A) Diagram depicting genomic region and P-element insertions within CG4164, which we have renamed shriveled (shv). Orange boxes indicate coding region of shv and gray boxes show untranslated mRNA. An adjacent gene, crq, is oriented in the opposite direction. Green boxes indicate crq coding region and darker gray boxes show untranslated mRNA. Blue inverse triangle indicates P-element insertions within the 5’-UTR of shv. SP represents signal peptide. A predicted nuclear localization signal (NLS) is also highlighted. Sequence alignment between human DNAJB11 and Shv are shown below. Red residues highlight the RGD sequence and the similar residues in fly. (B) Quantification of the relative shv and crq transcripts in control and shv1. * p < 0.05 compared to control. All values represent mean ± SEM. n = 4 independent experiments. For paired samples, Student’s T-test was used. (C) Shv antibody is specific for Shv. Western blot performed using fly extract detected with Shv antibody (control) and antibody pre-absorbed with Shv peptide. (TIF) [file pgen.1006043.s002.tif]

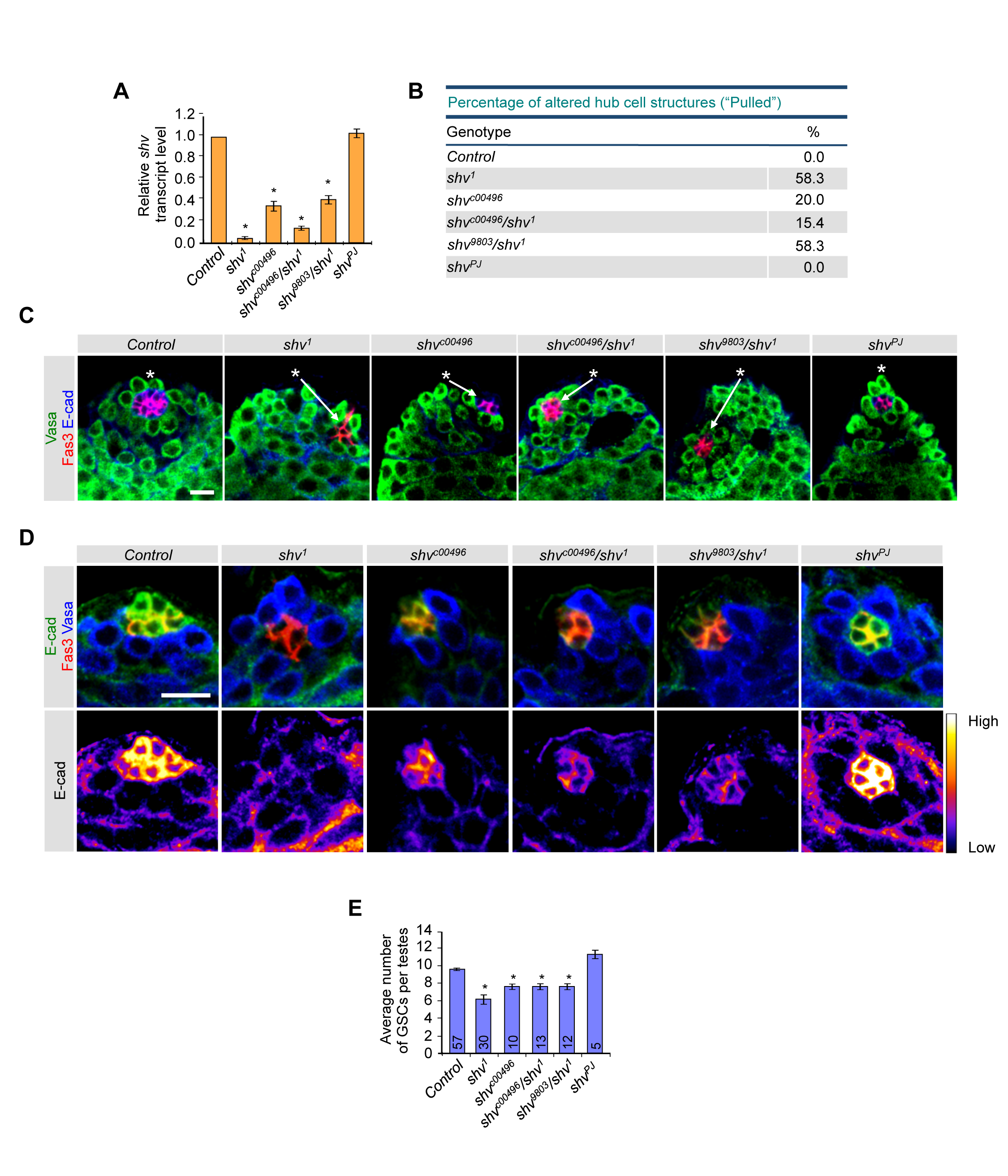

Supplement: S3 Fig — (A) Quantification of the relative shv transcripts. * p < 0.05 compared to control. All values represent mean ± SEM. n = 4 independent experiments. (B) Table showing percentages of testes with altered hub structure. (C) Representative images of testes dissected from 3-day old flies. Asterisks show the apical tip of the testis and arrows highlight distally located hub. (D) Testes stained with DE-cadherin (E-cad) and indicated antibodies. Lower panels show pseudo-colored images of DE-cadherin staining intensity. (E) Quantification of GSCs per testis dissected from 3-day old flies. * p < 0.05 compared to control. For multiple samples, One-way ANOVA followed by post hoc analysis with Bonferroni’s multiple-comparison test was used to determine statistical significance. Scale bars in (C) and (D) = 10 μm. (TIF) [file pgen.1006043.s003.tif]

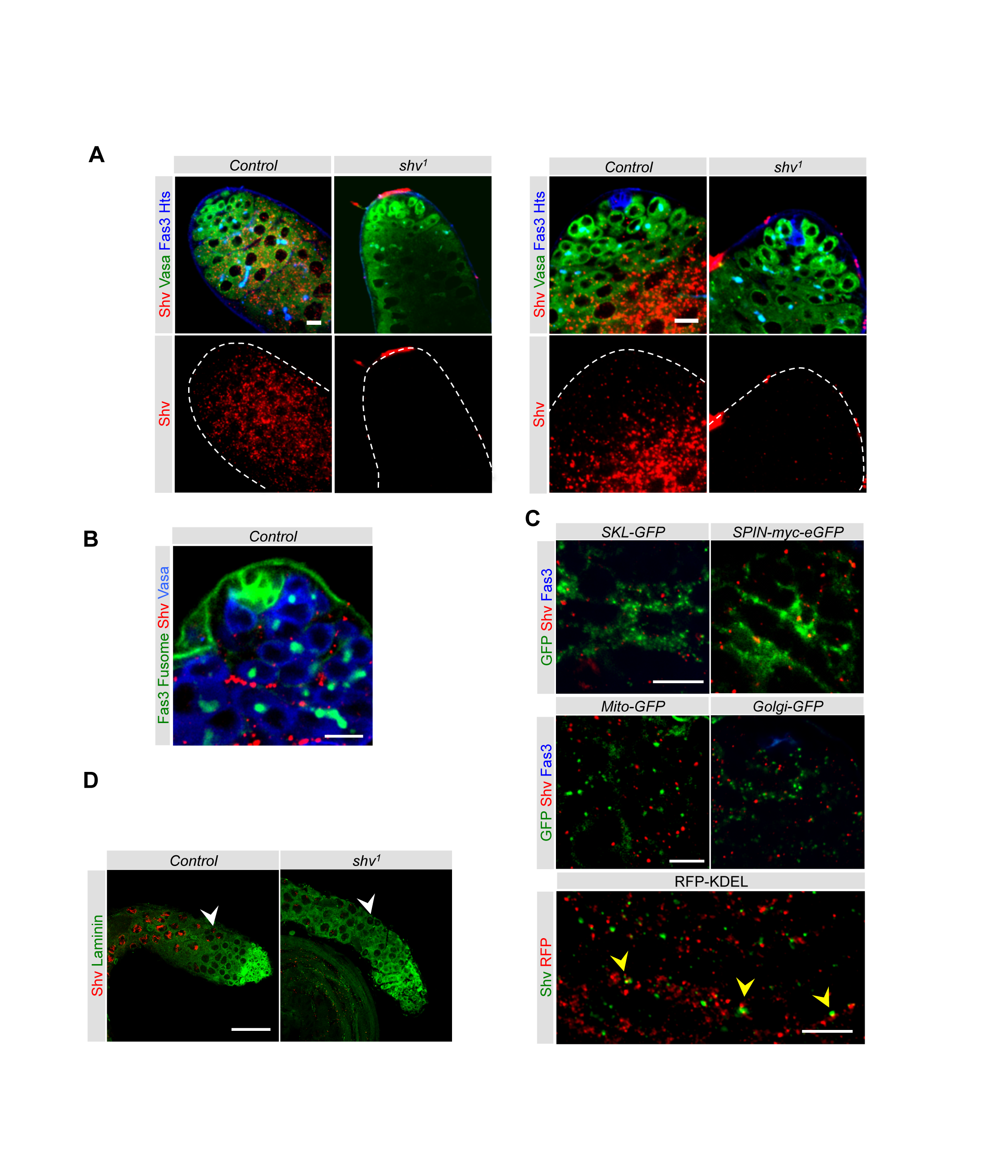

Supplement: S4 Fig — Dual fluorescent shv RNA and protein detection to mark different cell types. (A) Control and shv1 testes taken at lower magnification demonstrating ubiquitously expressed shv RNA at the apical tip of the testis. Higher magnification images representing the presence of shv RNA in the spermatocytes. shv RNA is seen in the hub, CySCs, germ and cyst cells of the control testes, but barely detectable in shv1 mutant. Similarly, shv RNA is seen at high level in control spermatocytes and cyst cells, but not in shv1 mutant. (B) Control testes stained with Shv, fusome and indicated antibody to see where Shv is located. (C) Shv subcellular distribution is further investigated by staining testes expressing fluorescently-tagged organelle markers with Shv antibody. Peroxisome marker (SKL-GFP); lysosme marker (SPIN-myc-eGFP); golgi marker (Golgi-GFP); mitochondria marker (mito-GFP); ER marker (RFP-KDEL). For (A), (B), (C), scale bar = 10 μm. (D) Abundant Shv protein is detected in the nucleus of control spermatocytes but is significantly reduced in shv1 (arrowhead). Scale bar = 50 μm. Age of flies examined is 3 days after eclosion. (TIF) [file pgen.1006043.s004.tif]

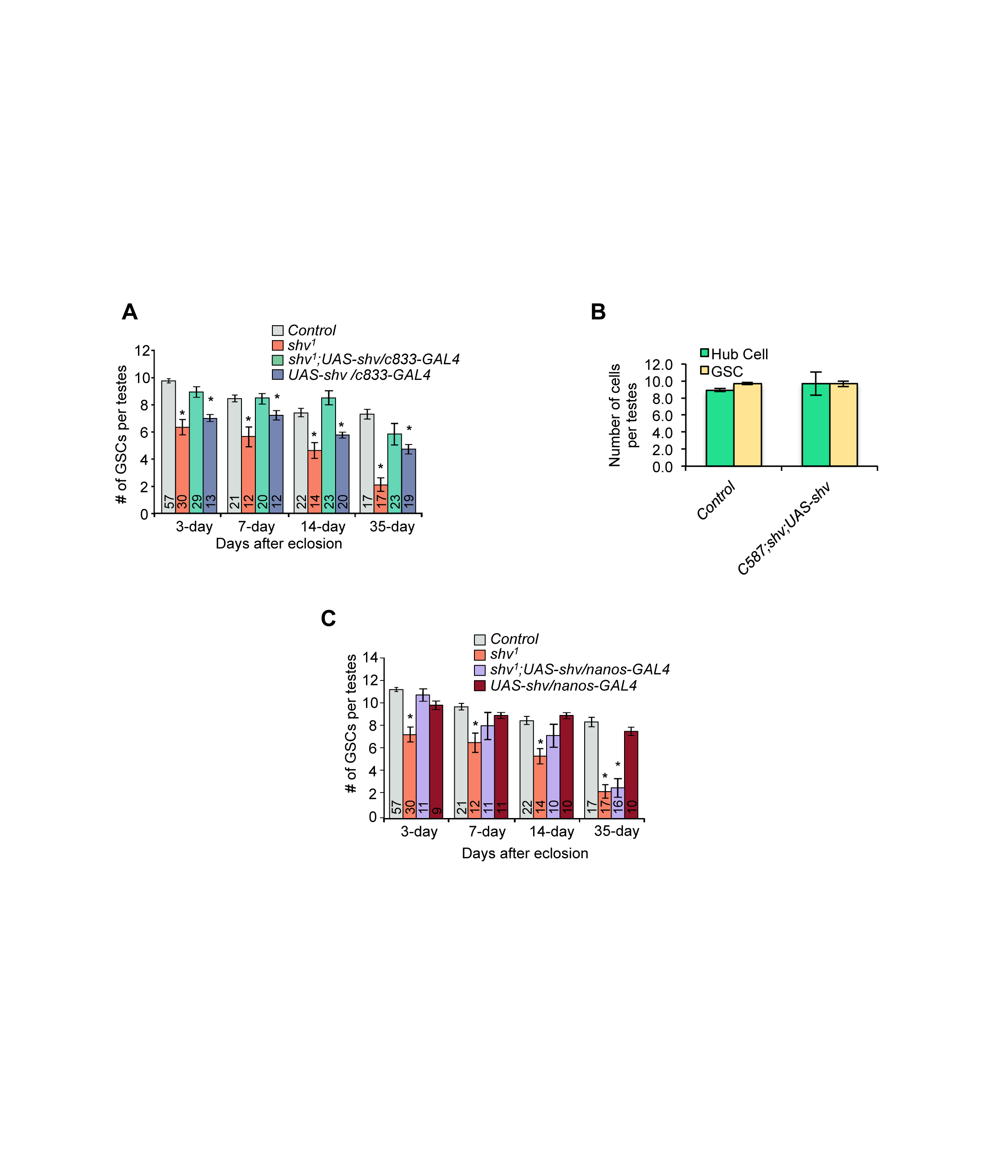

Supplement: S5 Fig — (A) Quantification of the average number of GSCs per testes for the indicated genotypes. (B) Quantification of the average number of hub and GSCs per testes for the indicated genotypes. (C) Quantification of the average number of GSCs per testes for the indicated genotypes. * p < 0.05 compared to control. For multiple samples, One-way ANOVA followed by post hoc analysis with Bonferroni’s multiple-comparison test was used to determine statistical significance. (TIF) [file pgen.1006043.s005.tif]

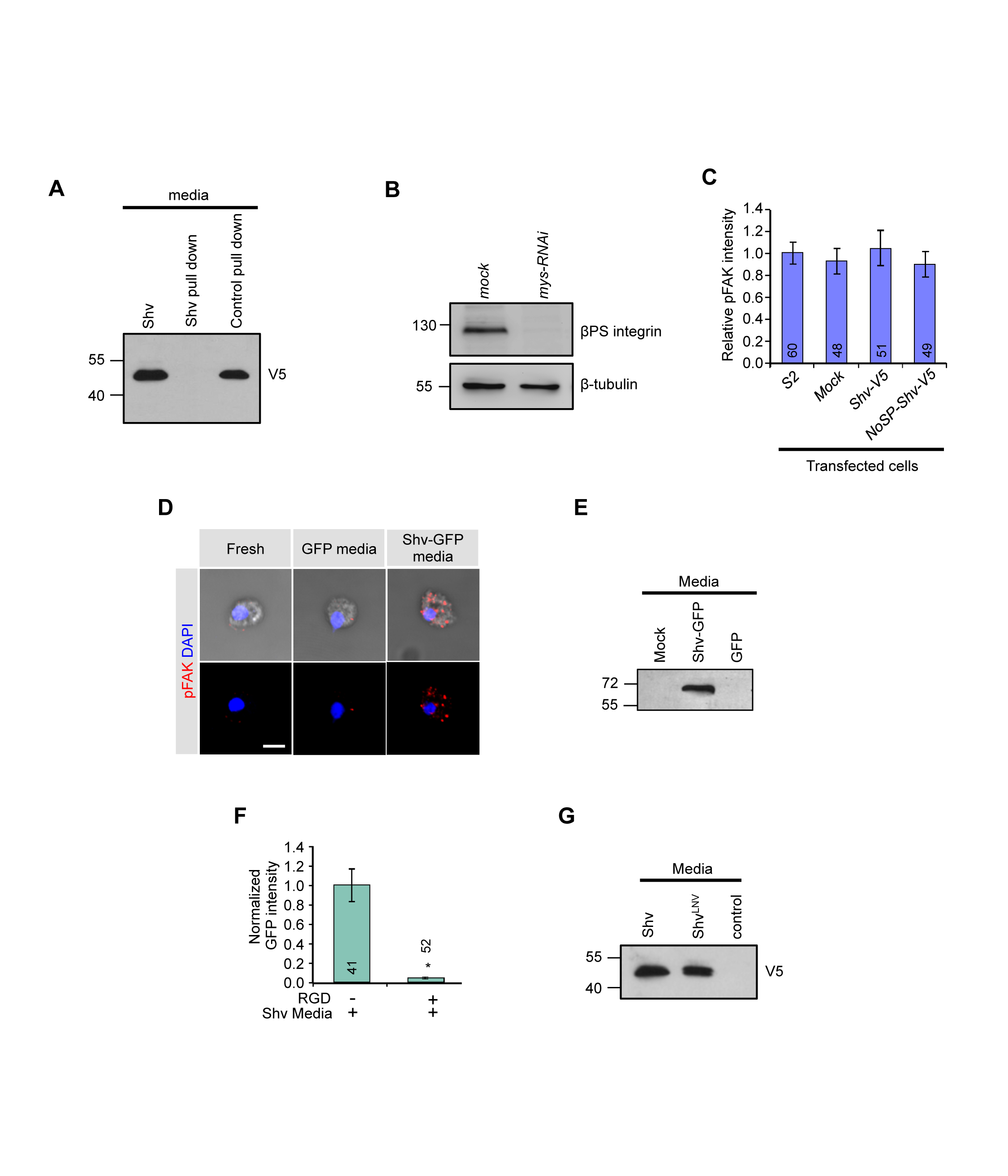

Supplement: S6 Fig — (A) Western blot depicting Shv levels in the media for the indicated conditions. Shv pull down efficiently removed Shv proteins from the media. (B) Western blot demonstrating efficiency of mys-RNAi. (C) Quantification of pFAK intensity in cells transfected with the indicated constructs. Intracellular expression of Shv did not alter pFAK levels when Shv was removed extracellularly. Number of cells examined is indicated in the bar graph. (D) Representative images of cell spreading and pFAK levels in S2 cells treated with the indicated media. (E) Western blot depicting the normal secretion of Shv protein tagged with GFP. (F) Quantification of GFP levels on the S2 cell surface normalized to the amount of GFP without RGD peptide incubation. * p < 0.05 compared to control. (G) Western blot demonstrating the presence of ShvLNV extracellular in the media. For paired samples, Student’s T-test was used. For multiple samples, One-way ANOVA followed by post hoc analysis with Bonferroni’s multiple-comparison test was used to determine statistical significance. Number of cells examined is indicated in the bar graph. All values represent mean ± SEM. Scale bar = 5 μm. (TIF) [file pgen.1006043.s006.tif]

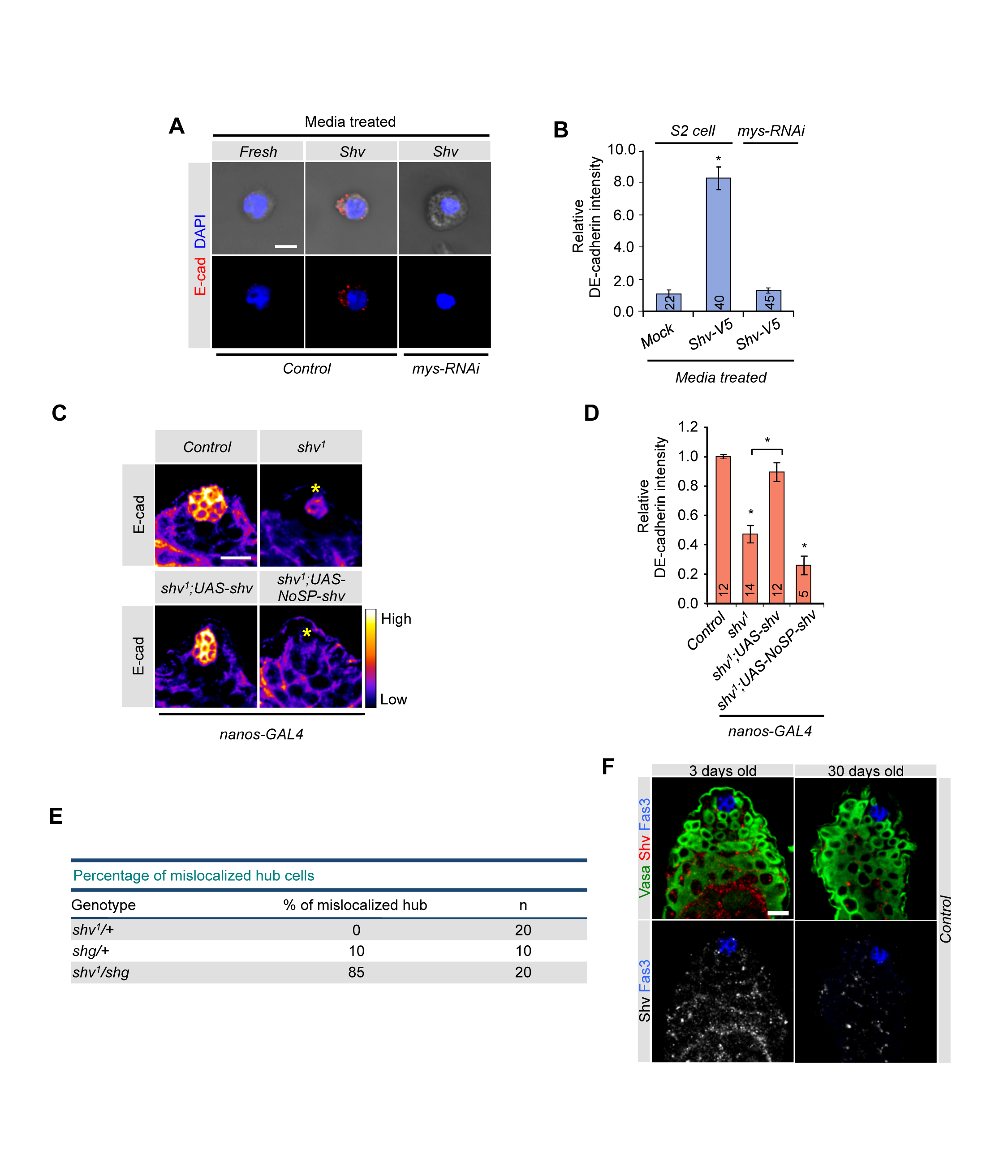

Supplement: S7 Fig — (A) Representative images of cell spreading and E-cad levels in S2 and mys-RNAi cells treated with the indicated media. Scale bar = 5 μm. (B) Quantification of E-cad intensity in control and mys-RNAi transfected cells following media treatment. (C) Pseudo-colored images of DE-cadherin in 3-day old testes. Asterisks indicate the hub. (D) Quantification of relative DE-cadherin intensity across genotypes. * p < 0.05 compared to control. ** p < 0.05 between the indicated genotypes. All values represent mean ± SEM. For multiple samples, One-way ANOVA followed by post hoc analysis with Bonferroni’s multiple-comparison test was used to determine statistical significance. n is indicated in the bar graph. (E) Percentage of mislocalized hub cells observed across genotype. (F) 3 and 30 days old testes labeled with Shv and indicated antibody. Scale bar in (C) and (F) = 10 μm. (TIF) [file pgen.1006043.s007.tif]
